# COVID-19 antibody responses in individuals with natural immunity and with vaccination-induced immunity: a systematic review and meta-analysis

**DOI:** 10.1186/s13643-024-02597-y

**Published:** 2024-07-19

**Authors:** Qiuying Zhang, Lirui Jiao, Qiushi Chen, Caroline A. Bulstra, Pascal Geldsetzer, Tulio de Oliveira, Juntao Yang, Chen Wang, Till Bärnighausen, Simiao Chen

**Affiliations:** 1 Chinese Academy of Medical Sciences and Peking Union Medical College, Beijing, China; 2 Department of Health Policy and Management, Gillings School of Global Public Health, University of North Carolina at Chapel Hill, Chapel Hill, NC USA; 3 The Harold and Inge Marcus Department of Industrial and Manufacturing Engineering, The Pennsylvania State University, University Park, PA USA; 4 Heidelberg Institute of Global Health, Faculty of Medicine and University Hospital, Heidelberg University, Im Neuenheimer Feld 130/3, Heidelberg, 69120 Germany; 5 Department of Public Health, Erasmus University Medical Center, Rotterdam, Netherlands; 6 Health Systems Innovation Lab, Harvard T.H. Chan School of Public Health, Harvard University, Cambridge, USA; 7 Division of Primary Care and Population Health, Stanford University, Stanford, CA USA; 8 Chan Zuckerberg Biohub, San Francisco, CA USA; 9 KwaZulu-Natal Research Innovation and Sequencing Platform, University of KwaZulu-Natal, Durban, South Africa; 10 Center for the AIDS Program of Research in South Africa (CAPRISA), Durban, South Africa

**Keywords:** COVID-19, Antibody response, Meta-analysis

## Abstract

**Background:**

The COVID-19 pandemic has caused a large mortality and morbidity burden globally. For individuals, a strong immune response is the most effective means to block SARS-CoV-2 infection. To inform clinical case management of COVID-19, development of improved vaccines, and public health policy, a better understanding of antibody response dynamics and duration following SARS-CoV-2 infection and after vaccination is imperatively needed.

**Methods:**

We systematically analyzed antibody response rates in naturally infected COVID-19 patients and vaccinated individuals. Specifically, we searched all published and pre-published literature between 1 December 2019 and 31 July 2023 using MeSH terms and “all field” terms comprising “COVID-19” or “SARS-CoV-2,” and “antibody response” or “immunity response” or “humoral immune.” We included experimental and observational studies that provided antibody positivity rates following natural COVID-19 infection or vaccination. A total of 44 studies reporting antibody positivity rate changes over time were included.

**Results:**

The meta-analysis showed that within the first week after COVID-19 symptom onset/diagnosis or vaccination, antibody response rates in vaccinated individuals were lower than those in infected patients (*p* < 0.01), but no significant difference was observed from the second week to the sixth month. IgG, IgA, and IgM positivity rates increased during the first 3 weeks; thereafter, IgG positivity rates were maintained at a relatively high level, while the IgM seroconversion rate dropped.

**Conclusions:**

Antibody production following vaccination might not occur as quickly or strongly as after natural infection, and the IgM antibody response was less persistent than the IgG response.

**Supplementary Information:**

The online version contains supplementary material available at 10.1186/s13643-024-02597-y.

## Introduction

Coronavirus disease 2019 (COVID-19), which is caused by severe acute respiratory syndrome coronavirus 2 (SARS-CoV-2), has exacted a massive global burden in terms of mortality and morbidity. As of 5 September 2023, there have been more than 695 million confirmed cases and nearly seven million deaths worldwide [1]. SARS-COV-2-infected individuals often suffer respiratory infections and may develop severe pneumonia, which can subsequently lead to multiple organ failure and death [2]. Although some antiviral medicines have been approved for the treatment of COVID-19 [3, 4], the natural immune response plays a vital role in patient recovery and in prevention of symptomatic disease [5].

SARS-CoV-2 is a member of the coronavirus family of viruses, which are transmitted mainly through respiratory droplets [6]. The mechanisms of SARS-CoV-2 infection and immunity depend on its simple biological structure, which consists of the hereditary material RNA with a nucleocapsid (N) protein, surrounded by an envelope [7]. The virus’s important structural proteins include the spike (S), envelope (E), and membrane (M) proteins [7, 8]. The S protein has two functional subunits, S1 and S2 [8]. SARS-CoV-2 utilizes the receptor-binding domain (RBD) on the S1 protein to bind to the angiotensin-converting enzyme 2 (ACE2) in infected individuals’ bodies, allowing it to enter host cells [7, 9, 10].

The term “immune response” refers to the body’s defenses against foreign components or mutated self-components. A significant aspect of human immunity involves the antibody response, referring to the secretion of antibodies that can bind to specific pathogen antigens, thereby preventing those pathogens from entering host cells [5]. Five different types of immunoglobulins—IgG, IgA, IgM, IgE, and IgD—work in different parts of the body, serving as antibodies. If a protein on a given pathogen can trigger an antibody response, that protein is considered immunogenic. For SARS-CoV-2, the RBD is highly immunogenic [11], and the S protein and N protein are also main immunogens [8, 12]. Antibodies are categorized as neutralizing or non-neutralizing depending on whether they can inhibit pathogen infectivity [13]. Neutralizing antibodies directly prevent infection by blocking interactions between pathogens and host-cell receptors [13]. With respect to SARS-CoV-2 infection, neutralizing activity has been reported in the immune system’s anti-RBD and anti-S responses [14].

Individual positivity for and levels of antibodies serve as good indicators for doctors and researchers who wish to study SARS-CoV-2 immunity, assess patient prognoses, and evaluate vaccine effectiveness. In most patients, SARS-CoV-2 triggers a detectable antibody response in the early phase of infection [15]. However, the protective capacity of the generated antibodies is not yet fully understood. Furthermore, some studies show that a proportion of SARS-CoV-2-positive individuals may be seronegative for antibodies [16–18].

Vaccination is one of the most effective public health tools for combatting COVID-19 and other infectious diseases [19]. As of 30 March 2023, a total of 183 COVID-19 vaccines had either been approved for use or were under clinical development [20], and additional vaccine candidates were in preclinical development [21]. Vaccination works by triggering the body’s immune response, and antibody response rates are good indicators to evaluate vaccine efficacy. For COVID-19, some studies have shown that the triggered antibody response lasts at least 3 months following vaccination [22]. However, other studies have indicated that the seroconversion rates for special immunoglobulins descend after a period, suggesting that vaccination-induced immunity is transient [5, 23].

A comprehensive understanding of the differences between artificial immunity and natural immunity would greatly contribute to the development of future vaccine products and vaccination programs. However, to date, few systematic reviews have been published to summarize existing knowledge of both vaccination-induced antibody responses and natural antibody responses for SARS-CoV-2. To fill this gap, we conducted a systematic review and meta-analysis to assess seroconversion rates over time under the natural antibody response (for both symptomatic and asymptomatic patients) and under the vaccination-induced antibody response.

## Methods

### Selection criteria

A pre-established protocol for this review is shown in Appendix File S2. We included experimental and observational studies that provide antibody positivity rates after natural COVID-19 infection or vaccination. Individuals in eligible studies were either qRT-PCR-diagnosed COVID-19 patients (including both symptomatic and asymptomatic cases) or vaccine recipients without documented past COVID-19 infection, were adults (aged 18 years or older), and had undergone serum antibody testing (for the neutralizing antibody, IgG, IgM, IgA, or total antibody) with at least 21 days’ follow-up. The main outcome was the seroconversion rate after a specific number of days following symptom onset/diagnosis, or after vaccination. The seroconversion rate was defined as the proportion of COVID-19 antibody seropositive participants to the total number of people tested. This study does not limit the types of antibodies detected, and studies reporting results for any specific antibody (the neutralizing antibody, IgG, IgM, IgA) or total antibody were considered for inclusion. Detection methods and timing of assessment were not restricted as well.

The exclusion criteria were the following: (1) We excluded reviews, comments, letters, patents, editorials, case reports, animal studies, conference abstracts, and studies of ten or fewer subjects. (2) We excluded articles focused on comparison of antibody testing techniques. (3) We excluded research conducted exclusively on special populations (i.e., studies in which all subjects either suffered the same comorbid disease such as HIV or diabetes, were healthcare workers, or were convalescent plasma donors) because the antibody response in these subjects may differ from the response in the general population. (4) We excluded studies to avoid population overlap of study subjects.

### Search strategy

We systematically searched PubMed, Web of Science, Embase, MEDLINE, medRxiv, and bioRxiv to identify all published and pre-publication studies with specified search terms between 1 December 2019 and 31 July 2023. Search terms included MeSH terms and “all field” terms comprising “COVID-19” or “SARS-CoV-2” and “antibody response” or “immunity response” or “humoral immune” (the detailed search terms for each database are shown in Appendix File S1). The reference lists of identified studies were also searched manually. The search was restricted to English language publications.

### Study selection

Two reviewers (QZ and LJ) independently screened each study’s title and abstract and examined full texts to assess article eligibility. Disagreement was resolved through discussion with a senior author (SC). This review has not been registered previously.

### Data extraction

The data extraction form used in this review was adapted from Cochrane’s data collecting form [24] and was shown in Appendix File S3. For the selected studies, we mainly collected the following information: author, publication year, region, sample size, sample mean or median age, gender composition, comorbidities, antibody testing method, antibody type, subject infection or vaccination status (including vaccine type and dose number), and median number of days to follow-up. To obtain the main outcome, we collected the number of seropositive participants, the total number of people tested, and the specific number of days following symptom onset/diagnosis, or after vaccination. Days following symptom onset/diagnosis/vaccination were divided into the following ranges: 0–7 days, 8–14 days, 15–21 days, 22 days–1 month, 1–2 months, 2–3 months, 3–6 months, and over 6 months. If the follow-up times reported in the article did not match these categories, we would try to obtain the original data and reclassify the serum results.

### Quality assessment

Since most of the included studies were observational or non-randomized experimental studies, the MINORS score (methodological index for non-randomized studies) was utilized, with an ideal score of 16 for non-comparative studies and 24 for comparative studies [25]. In this review, for non-comparative studies, a score of ≤ 10 would be considered poor quality, 11–14 as moderate quality, and 15–16 as good quality. For comparative studies, we considered a high-quality study to have ≥ 23 points and a low-quality study to have ≤ 16 points. As for the randomized studies, Risk of Bias 1 (ROB 1) tool was used to assess quality through the RevMan software version 5.3 [26].

### Heterogeneity

Between-study heterogeneity was assessed by the *Q* test, and the *I*^2^ statistic was calculated, with values of ≤ 50%, 50–75%, and > 75% indicating low, moderate, and high heterogeneity, respectively [27].

### Data analysis

Since the main purpose of the study is to explore the changes and differences in serum positivity rates, we used proportional meta-analysis as the statistical method for data analysis. Proportional meta-analysis is a statistical technique used to analyze and combine the results from multiple studies that report proportions or rates of a particular event or characteristic, like incidence or prevalence of disease [28, 29]. In this study, the proportion of interest is COVID-19 antibody serum positivity rate, which is defined as the number of seropositive participants divided by the total number of people tested. Studies were combined in meta-analyses using the random effect model because of substantial heterogeneity among studies [30]. For each analysis, we considered *p* values < 0.01 to be statistically significant (two-tailed). All analyses were performed using R software version 4.1.2 [31].

### Subgroup and sensitivity analysis

To compare the antibody response between SARS-CoV-2-infected individuals and vaccinees, a subgroup analysis was performed. We also conducted a subgroup analysis by antibody type (IgG, IgA, IgM, or neutralizing antibody) to further assess sources of heterogeneity.

To evaluate the robustness of our findings, a sensitivity analysis was performed by systematically excluding each study one at a time.

### Publication bias

In the assessment of publication bias, we employed funnel plots to visually inspect the asymmetry in the distribution of effect sizes. Furthermore, Egger’s regression test was conducted to quantitatively assess the presence of publication bias [32].

## Results

### Characteristics of the studies

**Fig. 1:**
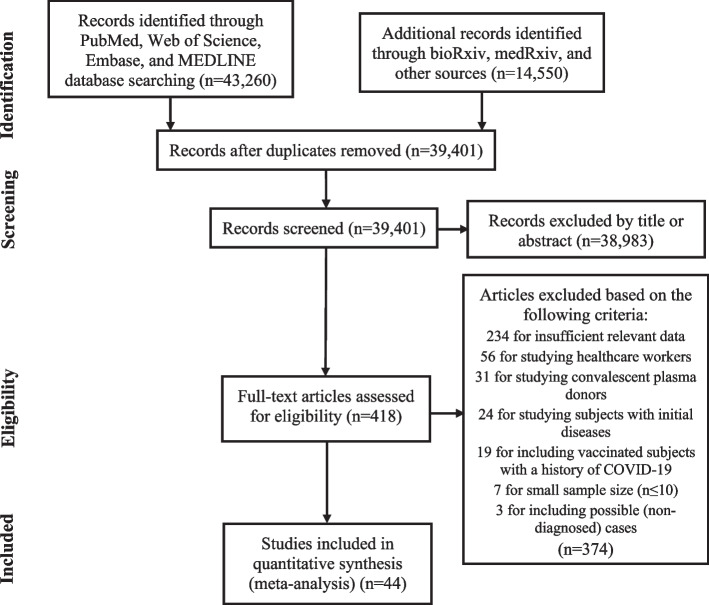
PRISMA flow chart of the selection procedure [33]

Of 15,656 studies from PubMed, 25,135 studies from Web of Science, 2469 studies from Ovid (containing Embase and MEDLINE), and 14,550 studies from bioRxiv and medRxiv, 418 studies were assessed for eligibility. After exclusion of articles according to different criteria, 44 studies were included in the final meta-analysis (Fig. 1). There are 40 observational studies in total, including 3 retrospective studies and 37 prospective studies (29 longitudinal studies, 2 cross-sectional studies, and 6 cohort studies). There are 4 experimental studies including 3 non-randomized studies and 1 randomized controlled trial.

Among these 44 included studies, 12 studies comprising a total of 43,664 subjects investigated antibody responses after vaccination with various types of COVID-19 vaccine (BNT162b2 [34–37], CoronaVac [23, 36, 38–40], ChAdOx1 [35–37], BBIBP-CorV [39, 41, 42], and mRNA-1237 [37]). Thirty-two studies comprising 9983 patients reported on antibody responses after infection. Median follow-up time was longer than 1 month for most studies, and the most common detection method were chemiluminescent immunoassay (50.0%) or enzyme-linked (43.2%). Basic information about the included studies is shown in Table 1 and the detailed demographic information is presented in Table S1.

**Table 1:** Basic characteristics of the included studies

Study	Study type	Detection method	Antibody type	Sample size	Types of participants	Vaccine type	Dose number	Follow-up days
Padoan et al. 2020 [43]	Prospective, longitudinal	CLIA; ELISA	IgA, IgM	70	Infection	–	–	23 days
Long et al. 2020 [17]	Prospective, cross-sectional	MCLIA	IgG, IgM	285	Infection	–	–	27 days
Mao et al. 2020 [44]	Prospective, longitudinal	MCLIA; MA	IgG, IgA, IgM, Nab	160	Infection	–	–	2 months
Ren et al. 2020 [45]	Retrospective	ELISA; MA	IgG, IgM, Nab	191	Infection	–	–	2 months
Jiang et al. 2020 [46]	Prospective, cohort	CLIA	IgG, IgM	214	Infection	–	–	2 months
Liu et al. 2020 [47]	Prospective, longitudinal	MCLIA	IgG, IgM	52	Infection	–	–	6 months
Solbach et al. 2020 [48]	Prospective, longitudinal	ELISA	IgG, IgA	118	Infection	–	–	2 months
Kowitdamrong et al. 2020 [49]	Prospective, longitudinal	ELISA	IgG, IgA	118	Infection	–	–	2 months
Maine et al. 2020 [50]	Prospective, cohort	MCLIA	IgG, IgM	427	Infection	–	–	3 months
Zhao et al. 2020 [51]	Prospective, longitudinal	ELISA	IgG, IgM, Ab	173	Infection	–	–	2 months
Li et al. 2020 [52]	Prospective, longitudinal	MCLIA	IgG, IgM	1850	Infection	–	–	3 months
Sterlin et al. 2021 [53]	Prospective, longitudinal	ELISA	IgG, IgA, IgM	132	Infection	–	–	1 month
Imai et al. 2021 [54]	Prospective, longitudinal	ELISA	IgG, IgM	231	Infection	–	–	1 month
Wu et al. 2021 [55]	Prospective, longitudinal	CLIA	IgG, IgM	349	Infection	–	–	7 months
Patil et al. 2021 [56]	Prospective, longitudinal	ELISA	IgG, IgA	66	Infection	–	–	22 days
Carnicelli et al. 2021 [57]	Prospective, cross-sectional	ELISA	IgG, IgA	131	Infection	–	–	2 months
Liu et al. 2021 [58]	Prospective, longitudinal	CLIA	IgG, IgM	1435	Infection	–	–	3 months
Kanedo et al. 2021 [34]	Non-randomized experimental	MCLIA	IgG	59	Vaccination	BNT162b2	1^a^	28 days
Yadav et al. 2021 [59]	Prospective, longitudinal	Not mention	IgG, IgM	1000	Infection	–	–	21 days
Brynjolfsson et al. 2021 [60]	Prospective, longitudinal	ELISA	IgG, IgA, IgM	221	Infection	–	–	28 days
Xiang et al. 2021 [61]	Prospective, longitudinal	CLIA	IgG, IgM	76	Infection	–	–	12 months
Luo et al. 2021 [62]	Prospective, longitudinal	MCLIA	IgG	20	Infection	–	–	11 months
Bueno et al. 2021 [23]	Randomized controlled trial	ELISPOT	IgG	270	Vaccination	CoronaVac	2	28 days
Wei et al. 2021 [35]	Retrospective	ELISA	IgG	23,368	Vaccination	ChAdOx1	1	14 weeks
				14,894		BNT162b2	1	
				1869		BNT162b2	1^a^	
Alshami et al. 2021 [63]	Prospective, longitudinal	CLIA	IgG	342	Infection	–	–	4 months
Akter et al. 2022 [64]	Prospective, longitudinal	ELISA	IgG, IgM	100	Infection	–	–	28 days
Bastug et al. 2022 [65]	Prospective, longitudinal	ELISA, VNA	IgG, IgM, Nab	129	Infection	–	–	28 days
Park et al. 2022 [66]	Prospective, longitudinal	ECLA	Ab	396	Infection	–	–	13 weeks
Yang et al. 2022 [67]	Prospective, longitudinal	ELISA, VNA	IgG, IgA, IgM, Nab	214	Infection	–	–	16 months
Barin et al. 2022 [36]	Prospective, longitudinal	Not mention	IgG	222	Vaccination	CoronaVac	2	3 months
				106		BNT162b2		
				56		ChAdOx1		
Cheng et al. 2022 [41]	Non-randomized experimental	CLIA	IgG, IgA, IgM	353	Vaccination	BBIBP-CorV	1^a^	6 months
Chen et al. 2022 [68]	Retrospective	CLIA	IgG, IgM	1093	Vaccination	Various types^b^	2	6 months
Chansaenroj et al. 2022 [69]	Prospective, cohort	ECLIA, VNA	IgG, IgA, Nab	531	Infection	–	–	12 months
Liang et al. 2022 [38]	Prospective, cohort	MCLIA	IgG, IgA, IgM, Nab	32	Vaccination	CoronaVac	1^a^	12 months
Kaduskar et al. 2022 [70]	Prospective, longitudinal	CLIA	IgG, IgM	138	Infection	–	–	4 months
Xu et al. 2022 [71]	Prospective, longitudinal	CLIA	IgG, IgM	34	Infection	–	–	12 months
Hua et al. 2022 [39]	Non-randomized experimental	CLIA	IgG, IgM, Nab	69	Vaccination	BBIBP-CorV	1^a^	7 months
				68		CoronaVac		
Wang et al. 2022 [42]	Prospective, cohort	VNA	Nab	275	Vaccination	WIBP-CorV + BBIBP-CorV	2	6 months
				133		BBIBP-CorV		
Ghasemi et al. 2022 [72]	Prospective, longitudinal	ELISA	IgG, IgM	98	Infection	–	–	6 months
Jager et al. 2022 [37]	Prospective, longitudinal	MCLIA	IgG	53	Vaccination	ChAdOx1	2	6 months
				28		BNT162b2	2	
				28		mRNA-1237	2	
Tao et al. 2022 [40]	Prospective, longitudinal	ELISA	IgG, IgM, Nab	93	Vaccination	CoronaVac	1^a^	2 months
Yuan et al. 2023 [73]	Prospective, longitudinal	ELISA	Nab	595	Vaccination	Inactivated vaccine	2	8 months
Bang et al. 2023 [74]	Prospective, longitudinal	ELISA	IgG	97	Infection	–	–	14 months
Carvalho et al. 2023 [75]	Prospective, cohort	CLIA	IgG	585	Infection	–	–	9 months

*Abbreviations*: *NA* not available, *IG* infection group—i.e., this study investigated antibody responses after infection instead of vaccination, *CLIA* chemiluminescent immunoassay, *ECLA* electrochemiluminescence assay, *ECLIA* electrochemiluminescence immunoassay, *ELISA* enzyme-linked immunosorbent assay, *ELISPOT* enzyme-linked immunospot assay, *MCLIA* microparticle chemiluminescent immunoassay, *MA* microneutralization assay, *VNA* virus-neutralizing assay

^a^Subjects received the second vaccine dose during follow-up

^b^Including BBIBP-CorV, CoronaVac, Zifivax, CanSino Ad5-nCoV, and Shenzhen Kangtai inactivated SARS-CoV-2 vaccine

### IgG response rates by vaccination and infection

**Fig. 2:**
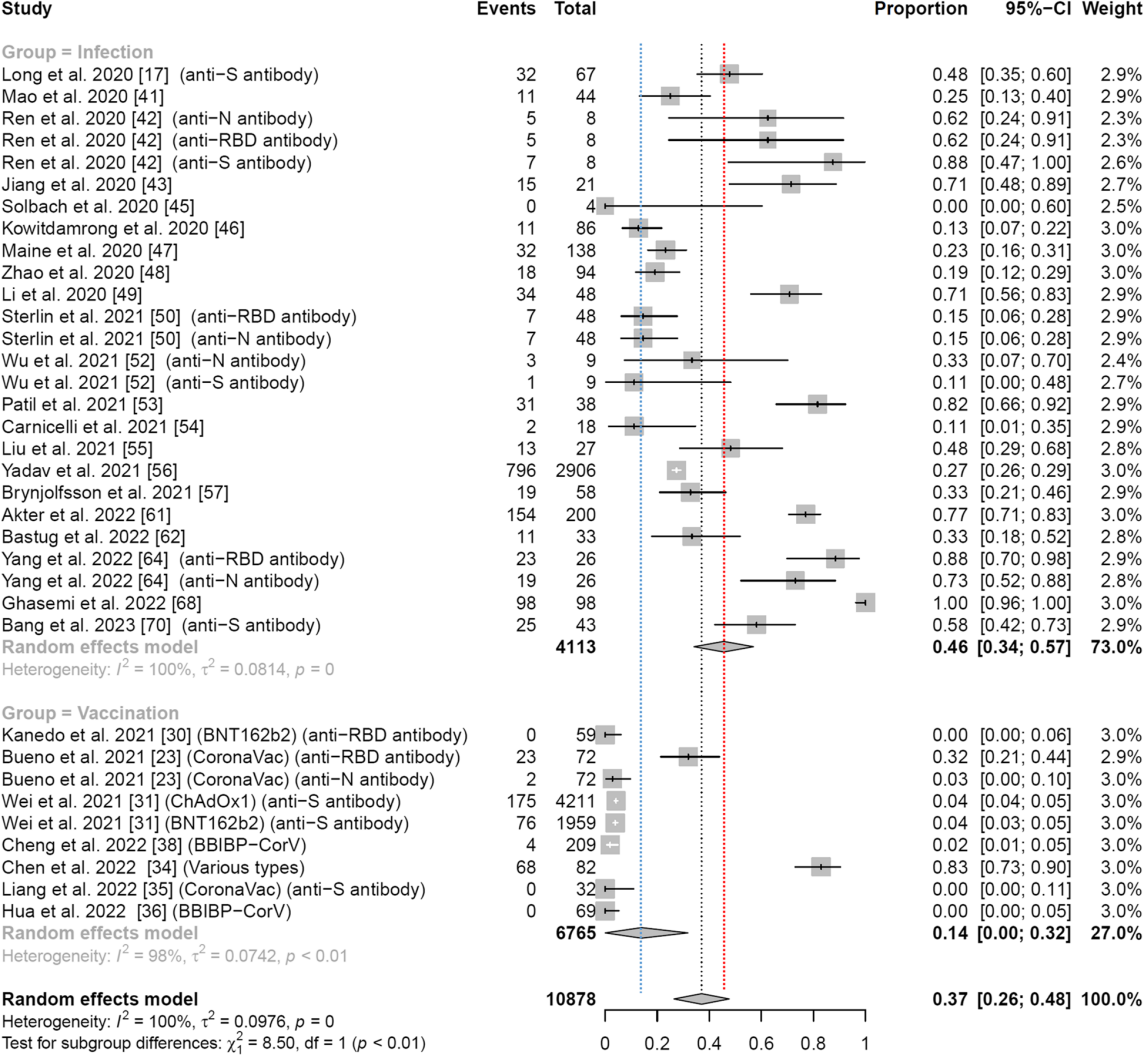
Forest plot of pooled IgG response rates during days 0–7 across vaccination and infection groups. Events: number of participants with detectable IgG antibody levels. Blue dashed line: pooled rate for vaccination group; red dashed line: pooled rate for infection group. The proportion of IgG-positive subjects is higher in the infection group than in the vaccination group (*p* < 0.01)

This subgroup analysis was performed to compare the antibody response between SARS-CoV-2-infected individuals and vaccinees. Twenty-eight studies comprising 10,878 subjects reported on the IgG antibody response in the first week following vaccination or COVID-19 symptom onset/diagnosis. The difference in antibody response between the vaccination group and the infection group was statistically significant (*p* < 0.01). In 7 studies of vaccinated subjects, 348 out of 6765 vaccinees showed a detectable IgG antibody response, and the pooled response rate was 0.14 (95% confidence interval [CI]: 0.00–0.32) with high heterogeneity (*I*^2^ = 98%). In 21 studies of naturally infected individuals, 1388 out of 4113 patients showed a detectable IgG antibody response, and the pooled response was 0.46 (95% CI: 0.34–0.57) with high heterogeneity (*I*^2^ = 100%). More details are given in Fig. 2. The results of this meta-analysis did not exhibit any statistically significant changes when systematically excluding each study one at a time and thus further reinforces the validity of our findings (Fig. S15).

**Fig. 3:**
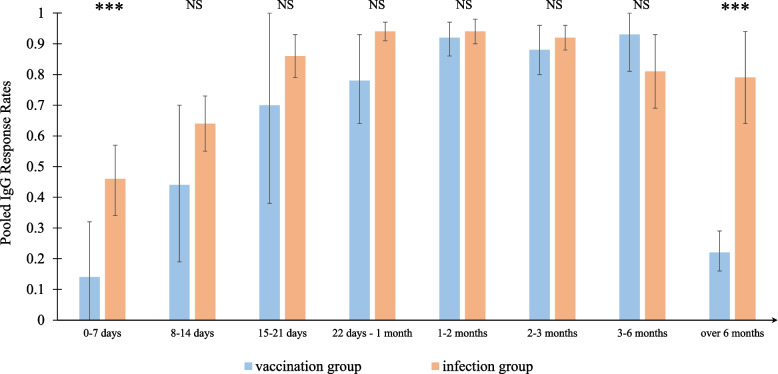
Pooled IgG antibody response rates across the vaccination group and infection group. ***: *p* < 0.01; NS: not significant

**Fig. 4:**
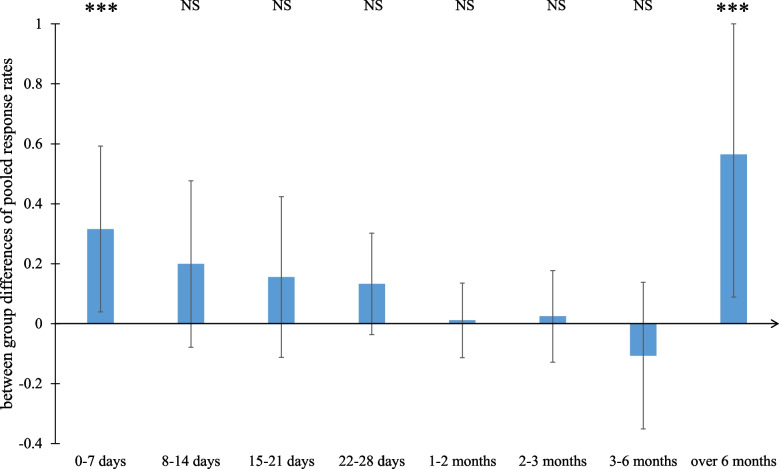
Between-group differences of the pooled IgG antibody response rates. ***: *p* < 0.01; NS: not significant

We further conducted subgroup analyses by time intervals of 0–7 days, 8–14 days, 15–21 days, 22 days–1 month, 1–2 months, 2–3 months, 3–6 months, and over 6 months. The stratified results are summarized in Fig. 3 (detailed forest plots for each subgroup by the time interval can be found in Figs. S1–S7 in the Appendix). Among the vaccination group, the pooled antibody response rates for the eight time intervals were 0.14, 0.44, 0.70, 0.78, 0.92, 0.88, 0.93, and 0.22, respectively; among the infection group, the pooled rates for the eight time intervals were 0.46, 0.64, 0.86, 0.94, 0.94, 0.92, 0.81, and 0.79, respectively. For both the infection and vaccination groups, the pooled IgG response rates were relatively low during the first week but increased later. The difference between the infection group and vaccination group was significant during the first week (*p* < 0.01), but any between-group differences were not significant from the second week to the sixth month (all *p* values > 0.01). However, after 6 months, the pooled IgG response rate of the vaccination group declined dramatically, and the difference between the infection group and the vaccination group was once again significant (*p* < 0.01). Comparing both the variables “Events” (number of individuals with positive serum) and “Total” (number of individuals tested), sizes of the between group differences were estimated and 95% confidence intervals were calculated using R package “metafor” [76], as shown in Fig. 4.

### Antibody response rates by antibody type

**Fig. 5:**
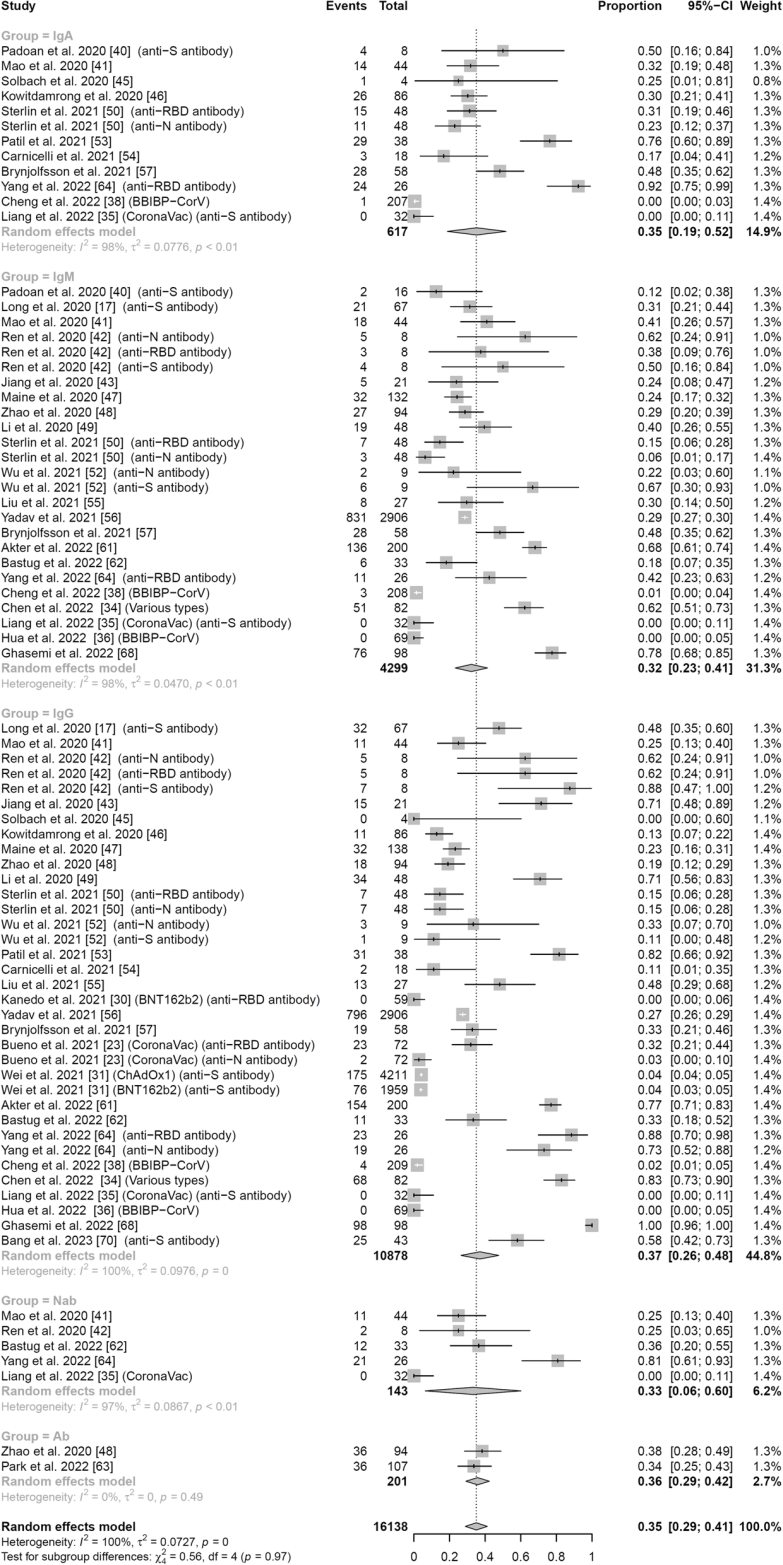
Forest plot of pooled antibody response rates 0–7 days following infection or vaccination by antibody type. Events: number of participants with detectable antibody levels. The differences in proportion of positive subjects by antibody type are non-significant (*p* = 0.97)

We then performed a subgroup analysis by antibody type (IgG, IgA, IgM, neutralizing antibody, and total antibody). Thirty studies comprising 16,138 subjects assessed antibody response rates 0–7 days following symptom onset or vaccination. No significant subgroup difference was identified for any antibody type (*p* = 0.97). For IgG, 1727 out of 10,878 subjects in 28 studies had detectable antibody levels, with a pooled response of 0.37 (95% CI: 0.26–0.48) and high heterogeneity (*I*^2^ = 100%). For IgM, 1304 out of 4299 subjects in 21 studies had detectable antibody levels, with a pooled response of 0.32 (95% CI: 0.23–0.41) and high heterogeneity (*I*^2^ = 98%). For IgA, 156 out of 617 subjects had detectable antibody levels, with a pooled response of 0.35 (95% CI: 0.19–0.52) and high heterogeneity (*I*^2^ = 98%). Finally, in the five studies that measured neutralizing antibody responses, 46 out of 143 subjects had detectable antibody levels, with a pooled response of 0.33 (95% CI: 0.06–0.60) and high heterogeneity (*I*^2^ = 97%). More detailed results can be found in the forest plot in Fig. 5. After excluding each study individually, the results of the meta-analysis remained stable, indicating the robustness and reliability of our study (Fig. S16).

**Fig. 6:**
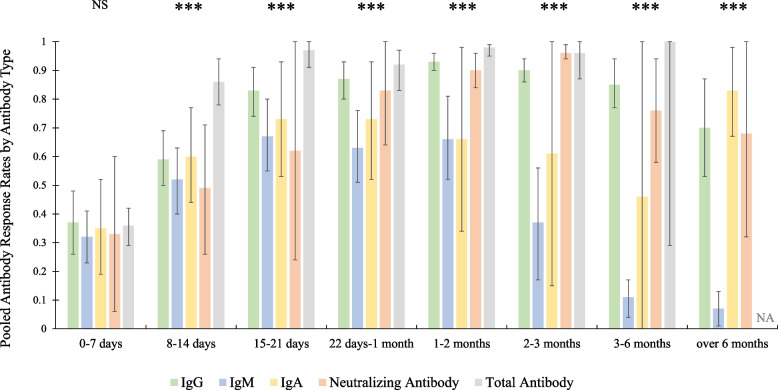
Pooled antibody response rates by antibody type. ***: *p* < 0.01; NS: not significant

For the antibody positivity rates at 8–14 days, 15–21 days, 22 days–1 month, 1–2 months, 2–3 months, 3–6 months, and after 6 months, the corresponding forest plots are provided in Figs. S8–S14 in the Appendix. The overall results are shown in Fig. 6. The pooled antibody response rates for IgG, IgM, IgA, neutralizing antibody, and total antibody increased from 0.37, 0.32, 0.35, 0.33, and 0.36, to 0.87, 0.63, 0.73, 0.83, and 0.92, respectively, during the first month. In the first week, between-group differences with respect to pooled antibody response rates were insignificant for all antibody types (*p* = 0.97). However, for follow-up during the second week and later, the between-group differences were significant (all *p* values < 0.01). The positivity rate for IgM dropped rapidly starting in the third month, with results of a hypothesis test indicating that this rate was significantly different from the positivity rate for IgG from this point onward (all *p* values < 0.01).

### Publication bias

The results of Egger’s regression test indicate that there is no significant evidence for the existence of publication bias (all *p* values > 0.01, Fig. S17).

## Discussion

This meta-analysis considered data from 44 studies of antibody response following COVID-19 vaccination or natural infection with SARS-CoV-2 (including both symptomatic and asymptomatic infection). To our knowledge, it is the first meta-analysis to assess antibody response rate changes over time when comparing vaccination-induced immunity and natural immunity, as well as different antibody types.

Our primary findings show that for both the vaccination group and the infection group, the pooled IgG antibody response rate increased continuously for the first month. In the first week, the pooled response rate was significantly higher for naturally infected patients than for vaccinees (*p* < 0.01), indicating that COVID-19 vaccination triggers a slower and more gentle IgG antibody response than natural infection. This suggests that existing vaccines have room for improvement respecting the speed with which they provide antibody protection. However, starting from the second week following symptom onset/diagnosis/vaccination, any differences between the infection group and the vaccination group considering IgG positivity rate were not significant (all *p* values > 0.01). This suggests that vaccination may match the effectiveness of natural infection in inducing antibody production after a short period, which would increase confidence in existing COVID-19 vaccines. However, after more than 6 months, the vaccination group’s IgG response rate declined sharply, suggesting the need for a booster dose.

For the analysis by antibody type, between-group differences were not significant during the first week following vaccination or infection (*p* = 0.97). The pooled responses for each antibody type were all relatively small during the first 7 days, and antibody positivity rates generally increased over the first month. Röltgen and Boyd drew a similar conclusion in a previous review [5]. Relatively high positivity rates for IgG and IgA appear to have been maintained after the first month, compared those observed for IgM. Moreover, after the second month, IgM positivity rates were significantly different from those for other antibody types, with the IgG response appearing to be stronger and more persistent. This is consistent with previous knowledge that IgM usually provides early-stage protection while IgG provides long-term immune memory [77, 78]. Additional sensitivity analyses conducted by excluding each study one at a time yielded similar results and supported our main findings.

These findings have several important implications for vaccine development. If the immune response to SARS-CoV-2 could be maintained at sufficiently high levels for a period of time, it would effectively block infection for that period, which could have significant benefits for suppressing COVID-19 transmission. Improving the speed and level of antibody response produced by vaccines could substantially improve the population antibody protection level. One potential means to accomplish this would be developing new vaccine adjuvants designed to extend the duration of the IgG antibody response. Our findings also have important implications for serological surveys, which can shed light on population immunity levels. For example, a positive IgM test may suggest early period of infection/post-vaccination, while a negative IgM test plus a positive IgG test may indicate that the antibody response has been sustained in vivo for a time.

A notable consideration is the cybersecurity of the research regarding the dynamics of antibody response following SARS-CoV-2 vaccination or infection. With the increasing reliance on digital healthcare systems for data collection and storage, it has become crucial to address potential cybersecurity issues. The COVID-19 pandemic has expedited the utilization of digital platforms for healthcare data management, encompassing the collection and analysis of antibody response data, the implementation of biological data mining and machine learning techniques [79], and the utilization of artificial intelligence (AI) [80]. However, the integration of digital systems also introduces potential vulnerabilities and cybersecurity risks. Several studies have proposed strategies to safeguard data, such as employing blockchain technology in public health, which can foster secure practices in the realm of SARS-CoV-2 antibody response research [81, 82].

Our study has several limitations. First, the number of included studies of vaccinated individuals was limited, and only several types of vaccines were included among all the World Health Organization-approved vaccines (Ad26.COV2.S was missing from the study). Thus, our results should be generalized with caution. Second, the antibody responses of individuals who have been naturally infected with COVID-19 could potentially be affected by any therapies to which they were exposed. Due to the lack of treatment information in most studies, we were unable to consider the impact of therapy on immunity outcomes. Subgroup analysis by patient hospitalization status might reveal the impact of treatment on antibody response rates if therapy information were accessible. Third, we observed high heterogeneity values. Subgroup and sensitivity analyses confirmed the robustness of the results; however, they did not unveil the sources of this high heterogeneity. Possible sources of heterogeneity include large variations in sample size, demographic differences, and antibody detection methods employed in the studies, among others. More analyses could be conducted to further explore sources of heterogeneity. Finally, while the seroconversion rate is a vital indicator for assessing antibody response, the strength of the antibody response (i.e., antibody amount) is another important dimension. Evaluating this aspect of the antibody response is a potential direction for future study.

## Conclusion

Vaccination may trigger a slower and gentler early antibody response against SARS-CoV-2 than natural infection, but seroconversion rates in vaccinees generally catch up after 1 week. IgG response is stronger and more persistent than the IgM response for both naturally infected and vaccinated individuals. These findings will provide useful insights on COVID-19 antibody responses and can help inform future vaccine development.

### Supplementary Information

Additional file 1: Appendix.

## Data Availability

The data will be made available from the corresponding authors upon reasonable request.
