# Supplementary material for: COVID-19 antibody responses in individuals with natural immunity and with vaccination-induced immunity: a systematic review and meta-analysis
Source: Syst Rev. 2024 Jul 19;13:189. doi: 10.1186/s13643-024-02597-y (PMC11264703; doi:10.1186/s13643-024-02597-y)

Appendix for

COVID-19 Antibody Responses in Individuals with Natural Immunity and with Vaccination-induced Immunity: A Systematic Review and Meta-analysis

This file includes:

File S1. Search strategy.

File S2. Review Protocol.

File S3. Data Extraction Form.

Table S1. Basic characteristics of the included studies.

Table S2. MINORS quality assessment of the non-randomized studies.

Table S3. Cochrane quality assessment of the randomized studies.

Table S4. PRISMA checklist.

Figure S1. (related to Figure 3) Forest plot of pooled IgG response rates at 8-14 days across vaccination and infection groups.

Figure S2. (related to Figure 3) Forest plot of pooled IgG response rates at 15-21 days across vaccination and infection groups.

Figure S3. (related to Figure 3) Forest plot of pooled IgG response rates at 22 days – 1 month across vaccination and infection groups.

Figure S4. (related to Figure 3) Forest plot of pooled IgG response rates at 1-2 months across vaccination and infection groups.

Figure S5. (related to Figure 3) Forest plot of pooled IgG response rates at 2-3 months across

vaccination and infection groups.

Figure S6. (related to Figure 3) Forest plot of pooled IgG response rates at 3-6 months across vaccination and infection groups.

Figure S7. (related to Figure 3) Forest plot of pooled IgG response rates after 6 months across vaccination and infection groups.

Figure S8. (related to Figure 5) Forest plot of pooled antibody response rates at 8-14 days across antibody types.

Figure S9. (related to Figure 5) Forest plot of pooled antibody response rates at 15-21 days across antibody types.

Figure S10. (related to Figure 5) Forest plot of pooled antibody response rates at 22 days -1 month across antibody type.

Figure S11. (related to Figure 5) Forest plot of pooled antibody response rates at 1-2 months across antibody type.

Figure S12. (related to Figure 5) Forest plot of pooled antibody response rates at 2-3 months across antibody type.

Figure S13. (related to Figure 5) Forest plot of pooled antibody response rates at 3-6 months across antibody type.

Figure S14. (related to Figure 5) Forest plot of pooled antibody response rates after 6 months across antibody type.

Figure S15. (related to Figure 2) Sensitivity analysis: excluding each study one at a time.

Figure S16. (related to Figure 4) Sensitivity analysis: excluding each study one at a time.

Figure S17. Funnel plots of publication bias and results of Egger's test.

File S1. Search strategy.

PubMed:

((COVID-19[MeSH Terms]) OR (SARS-CoV-2[MeSH Terms])) AND ((antibody response) OR (immunity response) OR (humoral immune))

Web of Science:

((TS=(COVID-19)) OR TS=(SARS-CoV-2)) AND ((TS=(antibody response)) OR (TS=(immunity response)) OR (TS=(humoral immune)))

Ovid (containing Embase and MEDLINE):

(COVID-19 or SARS-CoV-2).sh. and (antibody response or immunity response or humoral immune).af.

bioRxiv and medRxiv:

((COVID-19) OR (SARS-CoV-2)) AND ((antibody response) OR (immunity response) OR (humoral immune))

File S2. Review Protocol

Background:

Coronavirus disease 2019 (COVID-19), caused by severe acute respiratory syndrome coronavirus 2 (SARS-CoV-2), has spread globally and caused a huge loss. By now, vaccination is the most effective public health method against this infectious disease. However, as some recent studies show, vaccine effectiveness declines so rapidly that the average protection effectiveness drops for 87.9% to 48.1% in a period of 8 months (1). Differences of antibody responses to diverse types of vaccines has been reported (2,3), but few studies compare the vaccine-caused antibody responses with the virus-caused antibody responses. Here we want to research the differences between natural immunity response (includes responses in both symptomatic and asymptomatic patients) and artificial immunity response. We hope that this study will provide some inspire for vaccine development as well as policy strategies.

Objectives:

Our study aims to achieve the following objectives. To:

1. Compare the seropositivity levels of antibodies against SARS-CoV-2 in nature and artificial immunity responses.

2. Compare the overtime changes of antibody positive rates between the nature and artificial immunity responses.

Methods:

We want to review systematically using the key words 'COVID-19' or 'SARS-Cov-2' and 'antibody response' or 'immunity response' or 'humoral responses' to identify all the published and pre-publication studies. Then meta-analyses will be conducted in order to evaluate the association between antibody levels and immunity responses types (patients or vaccinee).

Expected Outcomes:

Main expected outcomes of our meta-analysis will include antibody seropositivity levels of different immunity types. According to a former study (4), the antibody outcomes will include seropositivity levels of pan-immunoglobulins, IgM, IgA, and IgG antibodies against SARS-CoV-2 nucleocapsid(N), and neutralising antibodies.

References:

[1] B. A. Cohn et al., SARS-CoV-2 vaccine protection and deaths among US veterans during 2021, Science (2021).

[2] Dashdorj, N.J. et al., Direct Comparison of Antibody Responses to Four SARS-CoV-2 Vaccines in Mongolia, Cell Host and Microbe, (2021).

[3] Yuan, Ping et al., Safety, tolerability, and immunogenicity of COVID-19 vaccines: a systematic review and meta-analysis, medRxiv (2020).

[4] He, Ren et al., Seroprevalence and humoral immune durability of anti-SARS-CoV-2 antibodies in Wuhan, China: a longitudinal, population-level, cross-sectional study, the Lancet (2021).

File S3. Data Extraction Form

Review title

Study ID

I. Cover Page

**Study title**	
**Date of publication**	
**Reference citation**	
**Study authors contact details**	
**Publication type**	
**Notes**	

II. Eligibility Criteria: for intervention study only

**Study characteristics**	**Eligibility criteria**	**Yes/No/Unclear**	**Location in text or source**
Type of study	RCT		
	Non-RCT		
Participants	Adults without documented past COVID-19 infection		
Type of intervention	COVID-19 vaccination		
Outcome measures	seroconversion rate after a specific number of days after vaccination.		
Include or exclude			
Reason for exclusion			
Notes			

III. Eligibility Criteria: for observational study only

**Study characteristics**	**Eligibility criteria**	**Yes/No/Unclear**	**Location in text or source**
Type of study			
Participants	qRT-PCR-diagnosed COVID-19 patients		

Outcome measures	seroconversion rate after a specific number of days following symptom onset/diagnosis		
Include or exclude			
Reason for exclusion			
Notes			

IV. Characteristics of included studies: methods

	**Descriptions in paper**	**Location in text or source**
Aim of study		
Design		
Unit of allocation		
Follow-up days		
Ethical approval		
Notes		

V. Characteristics of included studies: participants

	**Description in paper**	**Location in text or source**
Population description		
Setting		
Inclusion criteria		
Exclusion criteria		
Recruitment method		
Informed consent		
Withdrawals and exclusions		
Age		
Sex		
Race / ethnicity		
Co-morbidities		
Region		
Notes		

VI. Characteristics of included studies: intervention groups (for intervention study only)

	**Description in paper**	**Location in text or source**
Group name		
Description		
Intervention		

Type of vaccine		
Timing		
Co-interventions		
Compliance		
Notes		

VII. Characteristics of included studies: outcomes

	**Description in paper**	**Location in text or source**
Outcome name		
Outcome definition		
Unit of measurement		
Testing method		
Antibody type		
Sample size		
Notes		

**Time points measured**	**Number of serum positive**	**Number of total**	**seroconversion rate**	**Location in text or source**

VIII. Risk of bias assessment: for randomized study only

**domain**		**Risk of bias**	**Location in text**
Selection bias	Random sequence generation		
	Allocation concealment		
Performance bias	Blinding of participants and personnel		
Detection bias	Blinding of outcome assessment		
Attrition bias	Incomplete outcome data		
Reporting bias	Selective reporting		
Other bias	Anything else, ideally prespecified		
Notes			

IX. Risk of bias assessment: for non-randomized study only

Domain	Score	Location in text
A clearly stated aim		
Inclusion of consecutive patients		
Prospective collection of data		
Endpoints appropriate to the aim of the study		
Unbiased assessment of the study endpoint		
Follow-up period appropriate to the aim of the study		
Loss to follow up less than 5%		
Prospective calculation of the study size		
An adequate control group *		
Contemporary groups *		
Baseline equivalence of groups *		
Adequate statistical analysis *		
Notes		

* Additional Criteria for comparative study

Notes: The items are scored 0 (not reported), 1 (reported but inadequate) or 2 (reported and adequate).

Table S1. Demographic characteristics of the Included Studies.

Study	Location	Sample Size	Mean / Median Age	Male / Female	Comorbidities	Number with comorbidities
Padoan et al. 2020 [40]	Italy	70	mean: 66.7	52 / 18	NA	NA
Long et al. 2020 [17]	China	285	NA	158 / 127	Hypertension, CVD, Diabetes, Malignancy, etc	87 (30.5%)
Mao et al. 2020 [41]	China	160	median: 46	NA	NA	NA
Ren et al. 2020 [42]	China	191	median: 58	114 / 77	diabetes, CVD, malignancies	41 (21.5%)
Jiang et al. 2020 [43]	China	214	median: 51	86 / 128	Hypertension, Diabetes, etc	43 (20.0%)
Liu et al. 2020 [44]	China	52	median: 54	33 / 19	Hypertension, Diabetes, etc	22 (42.3%)
Solbach et al. 2020 [45]	Germany	118	NA	51 / 67	NA	NA
Kowitdamrong et al. 2020 [46]	Thailand	118	median: 38	47 / 69	NA	NA
Maine et al. 2020 [47]	USA	427	NA	NA	NA	NA
Zhao et al. 2020 [48]	China	173	median: 48	84 / 89	Hypertension, Diabetes, etc	41 (24.0%)
Li et al. 2020 [49]	China	1850	NA	NA	NA	NA
Sterlin et al. 2021 [50]	France	132	NA	NA	NA	NA
Imai et al. 2021 [51]	Japan	231	median: 49	138 / 93	NA	NA
Wu et al. 2021 [52]	China	349	NA	NA	NA	NA
Patil et al. 2021 [53]	India	66	NA	54 / 21	CVD, diabetes, hypertension, chronic lung diseases, etc	NA
Carnicelli et al. 2021 [54]	Italy	131	median: 64	102 /29	NA	NA
Liu et al. 2021 [55]	China	1435	median: 61	683 / 752	NA	NA
Kanedo et al. 2021 [30]	Japan	59	median: 41	14 /45	NA	19 (32.2%)
Yadav et al. 2021 [56]	India	1000	mean: 47.5	687 / 313	NA	NA
Brynjolfsson et al. 2021 [57]	Iceland	221	mean: 52.7	91 /130	NA	NA

Xiang et al. 2021 [58]	China	76	median: 60	35 / 41	CVD, diabetes, hypertension, etc	36 (47.4%)
Luo et al. 2021 [59]	UK	20	median: 34.5	13 / 7	NA	NA
Bueno et al. 2021 [23]	Chile	270	mean: 40.4	NA	Healthy	-
Wei et al. 2021 [31]	UK	40131	median: 64	17963 / 22168	NA	NA
Alshami et al. 2021 [60]	Saudi Arabia	342	NA	209 / 133	NA	NA
Akter et al. 2022 [61]	Banglade sh	100	median: 47	60 / 40	NA	NA
Bastug et al. 2022 [62]	Turkey	129	mean: 46.4	70 / 59	Hypertension, CVD, Diabetes, etc	40 (31.0%)
Park et al. 2022 [63]	Korea	396	mean: 62	217 / 179	NA	NA
Yang et al. 2022 [64]	China	214	median: 48	100 /114	NA	NA
Barin et al. 2022 [32]	Cyprus	222	median: 63.5	78 / 144	Autoimmune disease, thyroid disease, arthritis, etc	246 (64.1%)
		106	median: 68	52 / 54		
		56	median: 59	24 / 32		
Cheng et al. 2022 [38]	China	353	NA	106 / 247	Healthy	-
Chen et al. 2022 [34]	China	1093	median: 51	453 / 640	NA	NA
Chansaenroj et al. 2022 [65]	Thailand	531	mean: 37.7	269 / 262	NA	NA
Liang et al. 2022 [35]	China	32	median: 35	8 / 24	NA	NA
Kaduskar et al. 2022 [66]	India	218	NA	131 / 87	NA	NA
Xu et al. 2022 [67]	China	66	mean: 47.09	33 / 33	Hypertension, Chronic liver disease, etc	27 (47.5%)
Hua et al. 2022 [36]	China	137	median: 42	47 / 90	Chronic respiratory diseases, diabetes, CVD	NA
Wang et al. 2022 [39]	China	275	mean: 37.94	113 / 162	NA	17 (6.23%)
		133	mean: 37.48	45 / 88		8 (6.02%)
Ghasemi et al. 2022 [68]	Iran	98	mean: 49.67	60 / 38	NA	NA
Jager et al. 2022 [33]	Austria	109	mean: 42.8	63 / 46	NA	NA
Tao et al. 2022 [37]	China	93	median: 34	33 / 60	Hypertension	2 (2.1%)

Yuan et al. 2023 [69]	China	595	mean: 35	146 / 449	NA	NA
Bang et al. 2023 [70]	Korea	97	mean: 59.5	54 / 43	NA	NA
Carvalho et al. 2023 [71]	Portugal	585	mean: 47.9	140 / 445	Hypertension, CVD, Diabetes, etc	56 (10.8%)

Abbreviations:

NA: Not Available; CVD: Cardiovascular diseases.

Table S2. MINORS Quality Assessment of the non-randomized Studies [24].

	A clearly stated aim	Inclusion of consecutive patients	Prospective collection of data	Endpoints appropriate to the aim of the study	Unbiased assessment of the study endpoint	Follow-up period appropriate to the aim of the study	Loss to follow up less than 5%	Prospective calculation of the study size	An adequate control group *	Contemporary groups *	Baseline equivalence of groups *	Adequate statistical analyses *	Total Score	Quality
Padoan et al. 2020 [40]	2	1	1	2	2	2	0	0					10	Low
Long et al. 2020 [17]	2	1	2	2	2	2	0	0					11	Moderate
Mao et al. 2020 [41]	2	1	2	2	2	2	0	0					11	Moderate
Ren et al. 2020 [42]	2	2	2	2	2	2	1	1					14	Moderate
Jiang et al. 2020 [43]	2	1	2	2	2	2	0	0					11	Moderate
Liu et al. 2020 [44]	2	1	2	2	2	2	0	0					11	Moderate
Solbach et al. 2020 [45]	2	1	2	2	2	2	1	0					12	Moderate
Kowitdamrong et al. 2020 [46]	2	2	2	2	2	2	0	0					12	Moderate
Maine et al. 2020 [47]	2	1	2	2	2	2	0	0					11	Moderate
Zhao et al. 2020 [48]	2	2	2	2	2	2	2	0					14	Moderate
Li et al. 2020 [49]	2	2	2	2	2	2	0	0					12	Moderate
Sterlin et al. 2021 [50]	2	2	2	2	2	2	0	0					12	Moderate
Imai et al. 2021 [51]	2	2	2	2	2	2	0	0					12	Moderate
Wu et al. 2021 [52]	2	2	2	2	2	2	0	0					12	Moderate
Patil et al. 2021 [53]	2	1	2	2	2	2	0	0					11	Moderate
Carnicelli et al. 2021 [54]	2	2	2	2	2	2	0	0					12	Moderate
Liu et al. 2021 [55]	2	2	2	2	2	2	0	0					12	Moderate
Kanedo et al. 2021 [30]	2	2	2	2	2	2	2	0					14	Moderate
Yadav et al. 2021 [56]	2	2	2	2	2	2	1	0					13	Moderate
Brynjolfsson et al. 2021 [57]	2	2	2	2	2	2	0	0	1	0	2	1	16	Low
Xiang et al. 2021 [58]	2	2	1	2	2	2	0	0					11	Moderate
Luo et al. 2021 [59]	2	2	2	2	2	2	1	0					13	Moderate
Wei et al. 2021 [31]	2	1	2	2	2	2	1	0					12	Moderate
Alshami et al. 2021 [60]	2	1	2	2	2	2	0	0					11	Moderate
Akter et al. 2022 [61]	2	1	2	2	2	2	2	0					13	Moderate
Bastug et al. 2022 [62]	2	2	2	2	2	2	0	0					12	Moderate
Park et al. 2022 [63]	2	2	2	2	2	2	0	0					12	Moderate
Yang et al. 2022 [64]	2	1	2	2	2	2	0	0					11	Moderate
Barin et al. 2022 [32]	2	1	2	2	2	2	0	0					11	Moderate
Cheng et al. 2022 [38]	2	2	2	2	2	2	1	1					14	Moderate
Chen et al. 2022 [34]	2	2	2	2	2	2	0	0	2	2	2	1	19	Moderate
Chansaenroj et al. 2022 [65]	2	1	2	2	2	2	0	0					11	Moderate
Liang et al. 2022 [35]	2	2	1	2	2	2	0	0					11	Moderate
Kaduskar et al. 2022 [66]	2	1	1	2	2	2	0	0					10	Low
Xu et al. 2022 [67]	2	1	2	2	2	2	0	0	1	2	2	1	17	Moderate

Hua et al. 2022 [36]	2	1	2	2	2	2	0	0					11	Moderate
Wang et al. 2022 [39]	2	2	2	2	2	2	2	2					16	High
Ghasemi et al. 2022 [68]	2	2	2	2	2	2	0	1					13	Moderate
Jager et al. 2022 [33]	2	1	2	2	2	2	1	0					12	Moderate
Tao et al. 2022 [37]	2	1	2	2	2	2	2	1					14	Moderate
Yuan et al. 2023 [69]	2	2	2	2	2	2	2	0					14	Moderate
Bang et al. 2023 [70]	2	2	2	2	2	2	1	1					14	Moderate
Carvalho et al. 2023 [71]	2	2	2	2	2	2	0	1					13	Moderate

* Additional Criteria for comparative study

Notes: The items are scored 0 (not reported), 1 (reported but inadequate) or 2 (reported and adequate). For non-comparative studies, a score of <= 10 would be considered poor quality, 11-14 as moderate quality, and 15-16 as good quality. For comparative studies, a score of <= 16 would be considered poor quality, 17-22 as moderate quality, and 23-24 as good quality.

Table S3. Cochrane Quality Assessment of the randomized Studies [78].

	**Selection bias**		**Performance bias**	**Detection bias**	**Attrition bias**	**Reporting bias**	**Other bias**
	**Random sequence generation**	**Allocation concealment**	**Blinding of participants and personnel**	**Blinding of outcome assessment**	**Incomplete outcome data**	**Selective reporting**	**Anything else, ideally prespecified**
Bueno et al. 2021 [23]	low	low	high	unclear	low	low	low

Table S4. PRISMA checklist [29].

Section and Topic	Item #	Checklist item	Location where item is reported
**TITLE**			
Title	1	Identify the report as a systematic review.	Title
**ABSTRACT**			
Abstract	2	See the PRISMA 2020 for Abstracts checklist.	Abstract
**INTRODUCTION**			
Rationale	3	Describe the rationale for the review in the context of existing knowledge.	Introduction, paragraphs 1-5
Objectives	4	Provide an explicit statement of the objective(s) or question(s) the review addresses.	Introduction, paragraph 6
**METHODS**			
Eligibility criteria	5	Specify the inclusion and exclusion criteria for the review and how studies were grouped for the syntheses.	Methods, 'selection criteria'
Information sources	6	Specify all databases, registers, websites, organisations, reference lists and other sources searched or consulted to identify studies. Specify the date when each source was last searched or consulted.	Methods, 'search strategy'
Search strategy	7	Present the full search strategies for all databases, registers and websites, including any filters and limits used.	Methods, 'search strategy'<br>Appendix, File S1
Selection process	8	Specify the methods used to decide whether a study met the inclusion criteria of the review, including how many reviewers screened each record and each report retrieved, whether they worked independently, and if applicable, details of automation tools used in the process.	Methods, 'search strategy'
Data collection process	9	Specify the methods used to collect data from reports, including how many reviewers collected data from each report, whether they worked independently, any processes for obtaining or confirming data from study investigators, and if applicable, details of automation tools used in the process.	Methods, 'Data extraction and outcomes'
Data items	10a	List and define all outcomes for which data were sought. Specify whether all results that were compatible with each outcome domain in each study were sought (e.g. for all measures, time points, analyses), and if not, the methods used to decide which results to collect.	Methods, 'Data extraction and outcomes'
	10b	List and define all other variables for which data were sought (e.g. participant and intervention characteristics, funding sources). Describe any assumptions made about any missing or unclear information.	Methods, 'Data extraction and outcomes'
Study risk of bias assessment	11	Specify the methods used to assess risk of bias in the included studies, including details of the tool(s) used, how many reviewers assessed each study and whether they worked independently, and if applicable, details of automation tools used in the process.	Methods, 'Quality Assessment and Data analysis', paragraph 1-2
Effect measures	12	Specify for each outcome the effect measure(s) (e.g. risk ratio, mean difference) used in the synthesis or presentation of results.	Methods, 'Quality Assessment and Data analysis', paragraph 3
Synthesis methods	13a	Describe the processes used to decide which studies were eligible for each synthesis (e.g. tabulating the study intervention characteristics and comparing against the planned groups for each synthesis (item #5)).	N/A
	13b	Describe any methods required to prepare the data for presentation or synthesis, such as handling of missing summary statistics, or data conversions.	Methods, 'Quality Assessment and Data analysis', paragraph 3
	13c	Describe any methods used to tabulate or visually display results of individual studies and syntheses.	Methods, 'Quality Assessment and Data analysis', paragraph 3

Section and Topic	Item #	Checklist item	Location where item is reported
	13d	Describe any methods used to synthesize results and provide a rationale for the choice(s). If meta-analysis was performed, describe the model(s), method(s) to identify the presence and extent of statistical heterogeneity, and software package(s) used.	Methods, 'Quality Assessment and Data analysis', paragraph 2-3
	13e	Describe any methods used to explore possible causes of heterogeneity among study results (e.g. subgroup analysis, meta-regression).	Methods, 'Quality Assessment and Data analysis', paragraph 3
	13f	Describe any sensitivity analyses conducted to assess robustness of the synthesized results.	Methods, 'Quality Assessment and Data analysis', paragraph 3
Reporting bias assessment	14	Describe any methods used to assess risk of bias due to missing results in a synthesis (arising from reporting biases).	Methods, 'Publication bias'
Certainty assessment	15	Describe any methods used to assess certainty (or confidence) in the body of evidence for an outcome.	Methods, 'Quality Assessment and Data analysis', paragraph 3
**RESULTS**			
Study selection	16a	Describe the results of the search and selection process, from the number of records identified in the search to the number of studies included in the review, ideally using a flow diagram.	Results, 'Characteristics of the Studies', paragraph 1
	16b	Cite studies that might appear to meet the inclusion criteria, but which were excluded, and explain why they were excluded.	N/A
Study characteristics	17	Cite each included study and present its characteristics.	Results, 'Characteristics of the Studies'<br>Table 1<br>Appendix Table S1
Risk of bias in studies	18	Present assessments of risk of bias for each included study.	Table 1<br>Appendix Table S2
Results of individual studies	19	For all outcomes, present, for each study: (a) summary statistics for each group (where appropriate) and (b) an effect estimate and its precision (e.g. confidence/credible interval), ideally using structured tables or plots.	Figure 2<br>Figure 4<br>Figure S1-S14
Results of syntheses	20a	For each synthesis, briefly summarise the characteristics and risk of bias among contributing studies.	N/A
	20b	Present results of all statistical syntheses conducted. If meta-analysis was done, present for each the summary estimate and its precision (e.g. confidence/credible interval) and measures of statistical heterogeneity. If comparing groups, describe the direction of the effect.	Results,'IgG Response Rates by Vaccination and Infection' and 'Antibody Response Rates by Antibody Type'
	20c	Present results of all investigations of possible causes of heterogeneity among study results.	Results,'IgG Response Rates by Vaccination and Infection' and 'Antibody Response Rates by Antibody Type'
	20d	Present results of all sensitivity analyses conducted to assess the robustness of the synthesized results.	Figure S15-16
Reporting biases	21	Present assessments of risk of bias due to missing results (arising from reporting biases) for each synthesis assessed.	Results, 'Publication bias'
Certainty of	22	Present assessments of certainty (or confidence) in the body of evidence for each outcome assessed.	Results,'IgG Response Rates

Section and Topic	Item #	Checklist item	Location where item is reported
evidence			by Vaccination and Infection' and 'Antibody Response Rates by Antibody Type'
**DISCUSSION**			
Discussion	23a	Provide a general interpretation of the results in the context of other evidence.	Discussion, paragraph 2-3
	23b	Discuss any limitations of the evidence included in the review.	Discussion, paragraph 5
	23c	Discuss any limitations of the review processes used.	Discussion, paragraph 5
	23d	Discuss implications of the results for practice, policy, and future research.	Discussion, paragraph 5
**OTHER INFORMATION**			
Registration and protocol	24a	Provide registration information for the review, including register name and registration number, or state that the review was not registered.	Methods, 'search strategy'
	24b	Indicate where the review protocol can be accessed, or state that a protocol was not prepared.	Appendix, 'file S2'
	24c	Describe and explain any amendments to information provided at registration or in the protocol.	Appendix, 'file S2'
Support	25	Describe sources of financial or non-financial support for the review, and the role of the funders or sponsors in the review.	Declarations, 'funding'
Competing interests	26	Declare any competing interests of review authors.	Declarations, 'Competing interests'
Availability of data, code and other materials	27	Report which of the following are publicly available and where they can be found: template data collection forms; data extracted from included studies; data used for all analyses; analytic code; any other materials used in the review.	Declarations, 'Availability of data and materials'

From: Page MJ, McKenzie JE, Bossuyt PM, Boutron I, Hoffmann TC, Mulrow CD, et al. The PRISMA 2020 statement: an updated guideline for reporting systematic reviews. BMJ 2021;372:n71. doi: 10.1136/bmj.n71

For more information, visit: http://www.prisma-statement.org/

Figure S1. (related to Figure 3) Forest plot of pooled IgG response rates at 8-14 days across vaccination and infection groups.

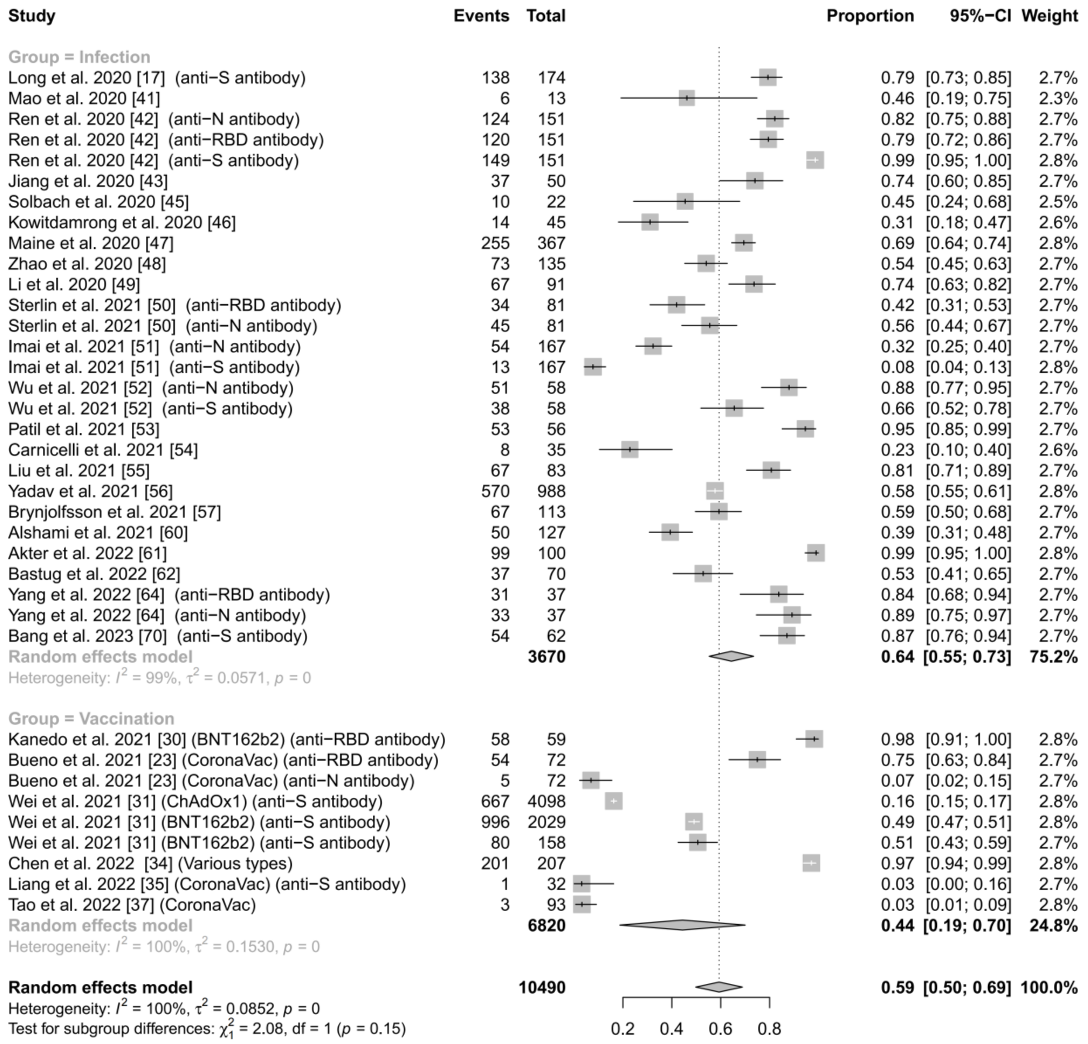

Events: number of participants with detectable IgG antibody levels.

Figure S2. (related to Figure 3) Forest plot of pooled IgG response rates at 9-21 days across vaccination and infection groups.

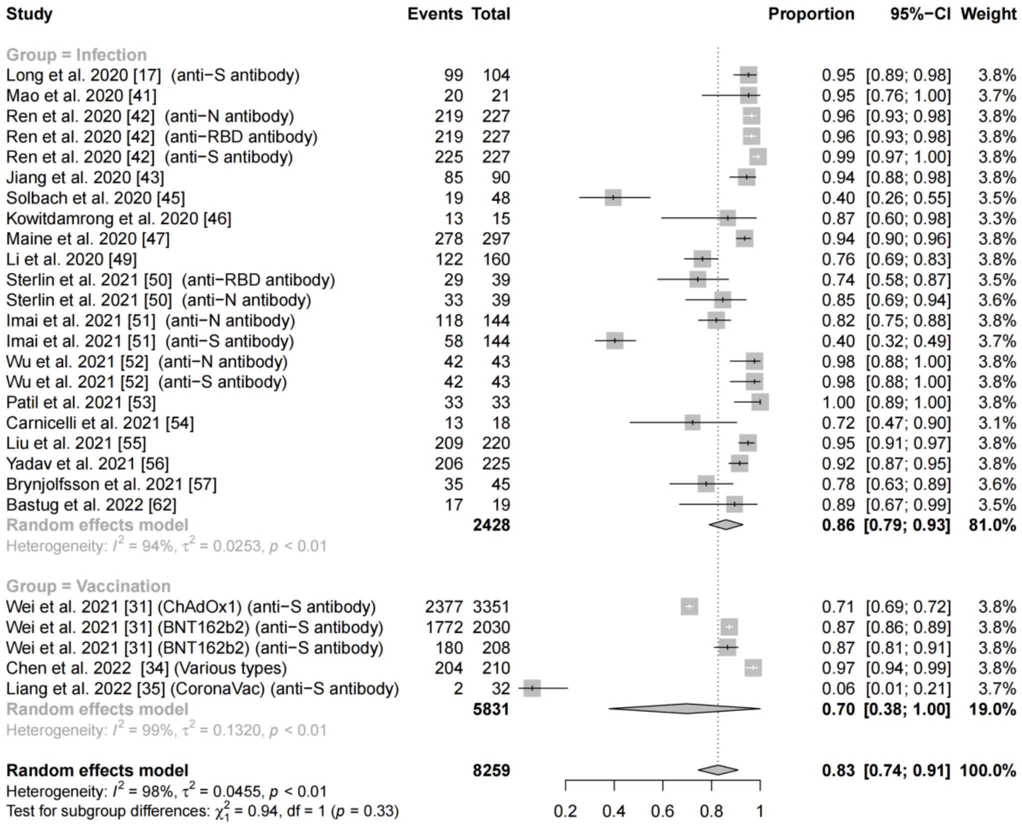

Events: number of participants with detectable IgG antibody levels.

Figure S3. (related to Figure 3) Forest plot of pooled IgG response rates at 22 days – 1 month across vaccination and infection groups.

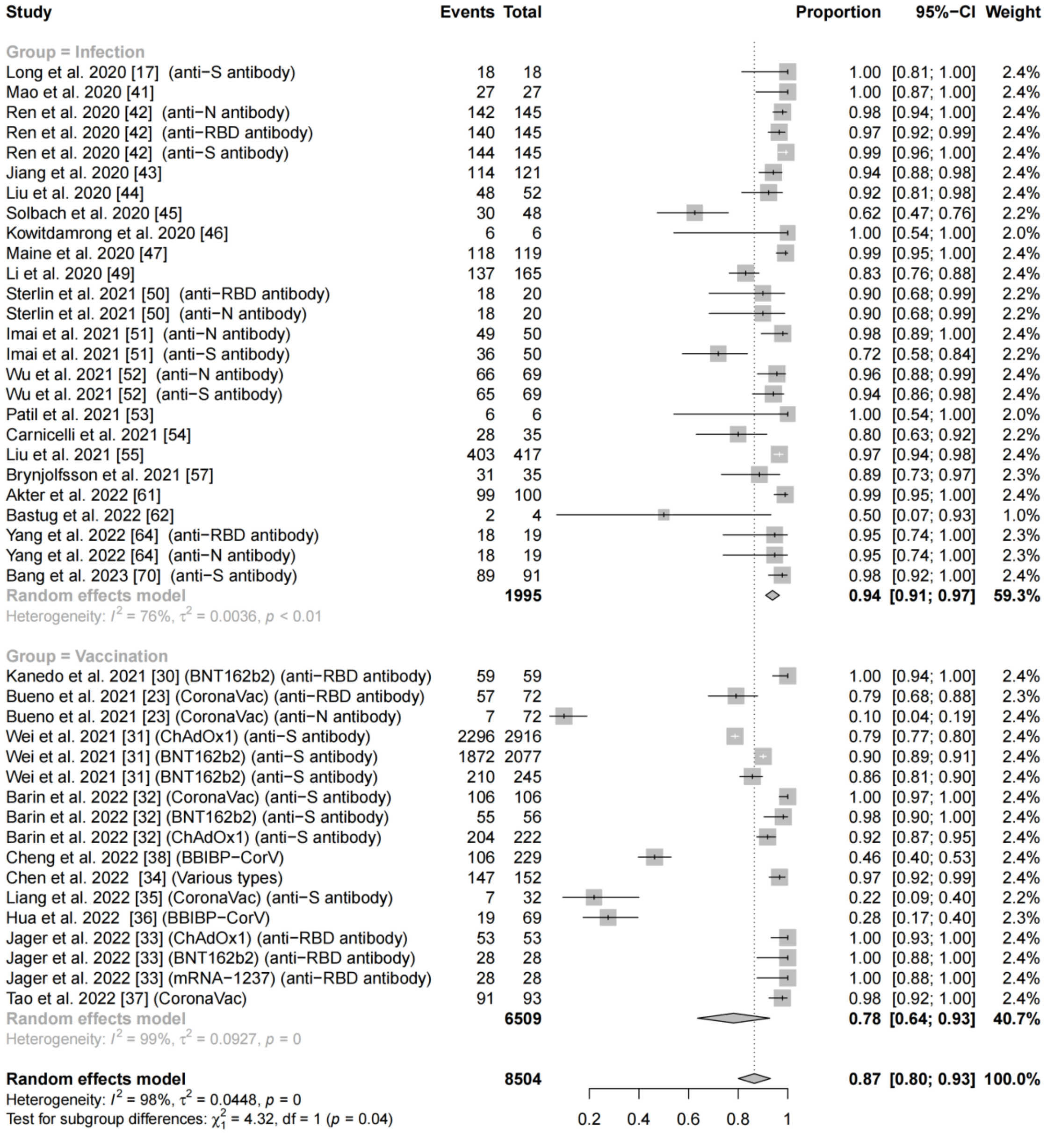

Events: number of participants with detectable IgG antibody levels.

Figure S4. (related to Figure 3) Forest plot of pooled IgG response rates at 1-2 months across vaccination and infection groups.

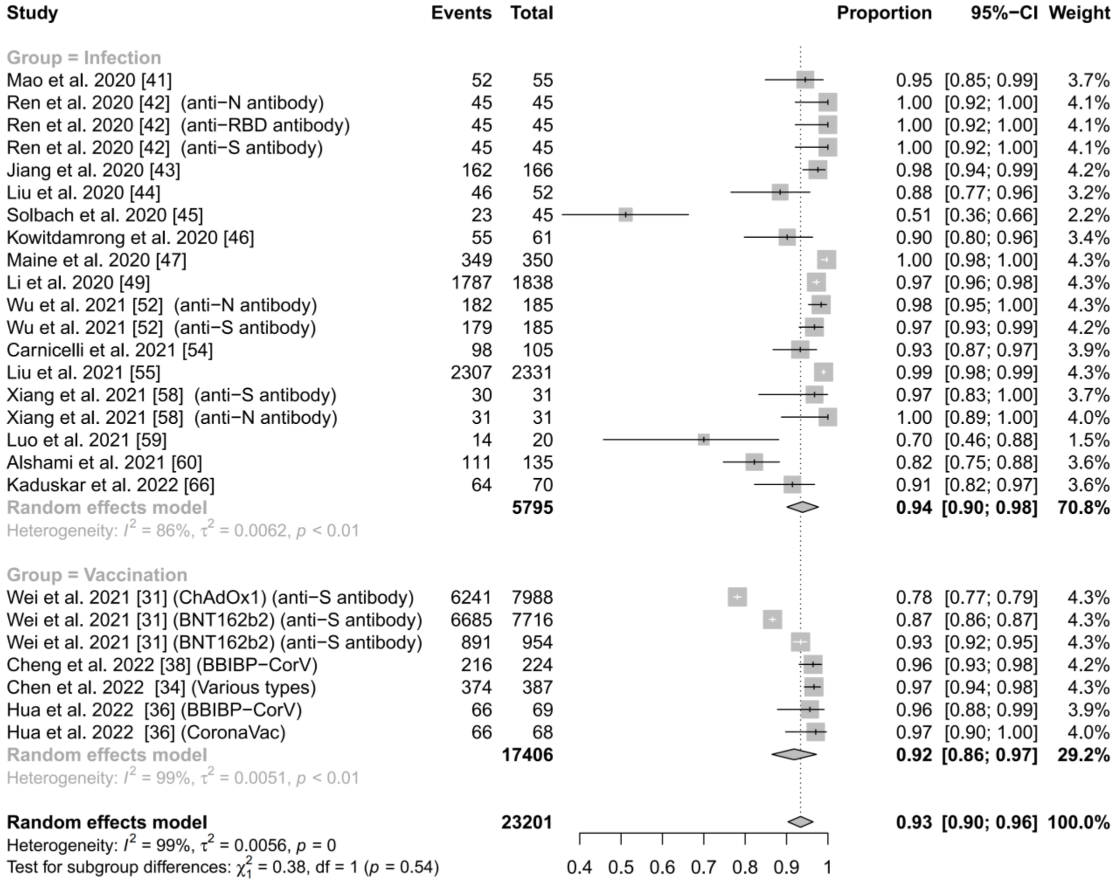

Events: number of participants with detectable IgG antibody levels.

Figure S5. (related to Figure 3) Forest plot of pooled IgG response rates at 2-3 months across vaccination and infection groups.

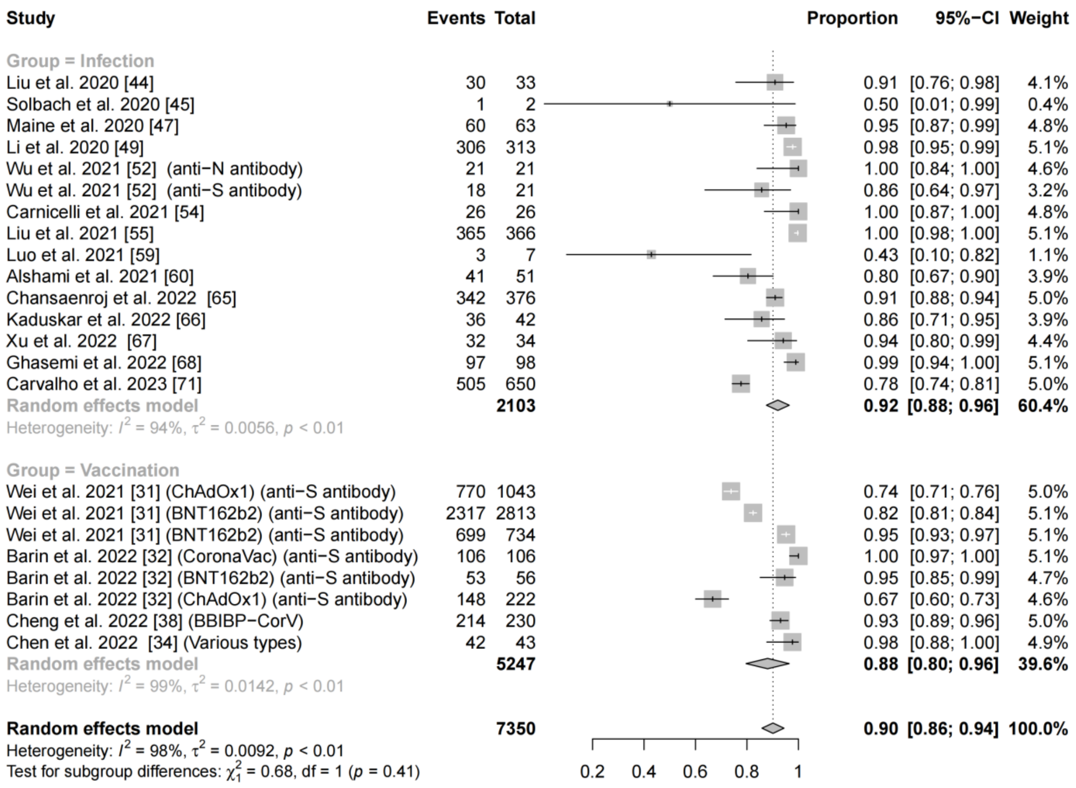

Events: number of participants with detectable IgG antibody levels.

Figure S6. (related to Figure 3) Forest plot of pooled IgG response rates at 3-6 months across vaccination and infection groups.

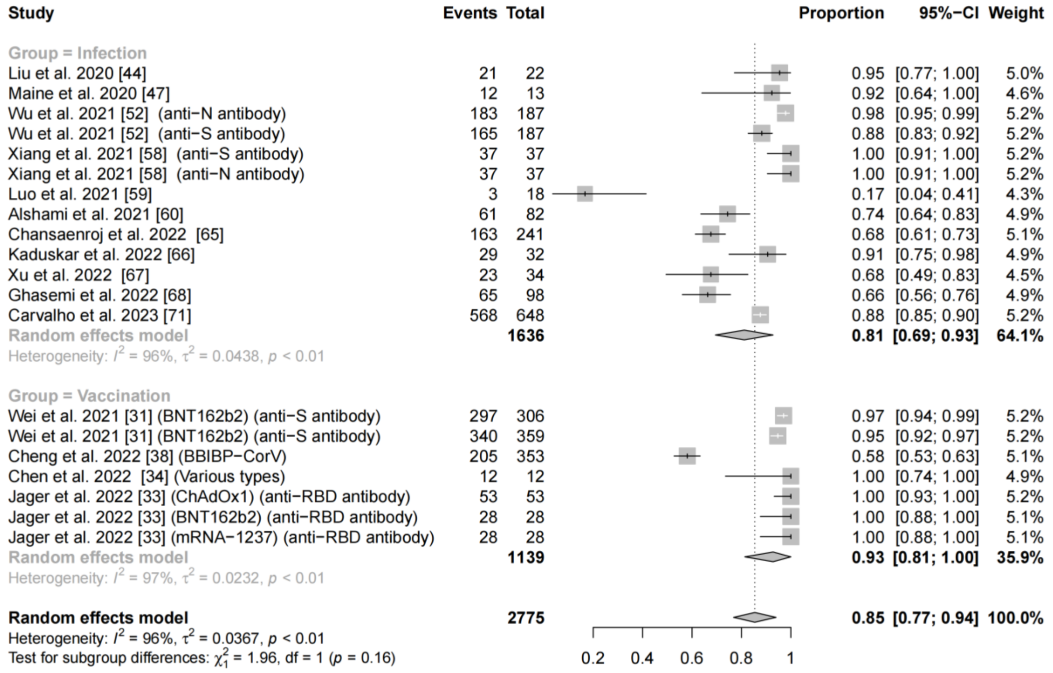

Events: number of participants with detectable IgG antibody levels.

Figure S7. (related to Figure 3) Forest plot of pooled IgG response rates after 6 months across vaccination and infection groups.

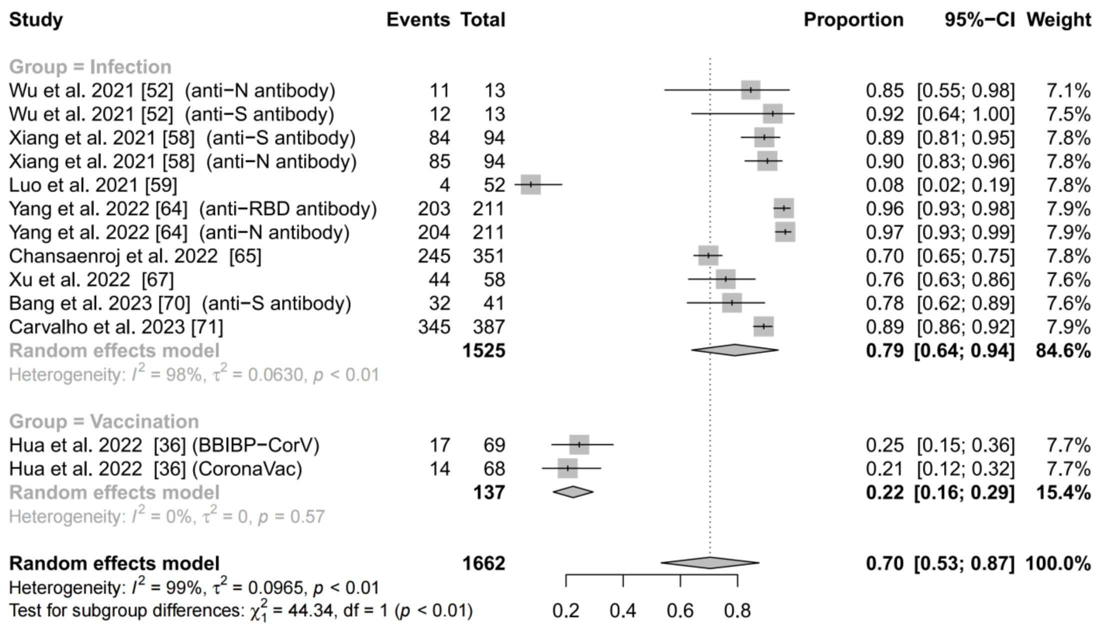

Events: number of participants with detectable IgG antibody levels.

Figure S8. (related to Figure 5) Forest plot of pooled antibody response rates at 8-14 days across antibody types.

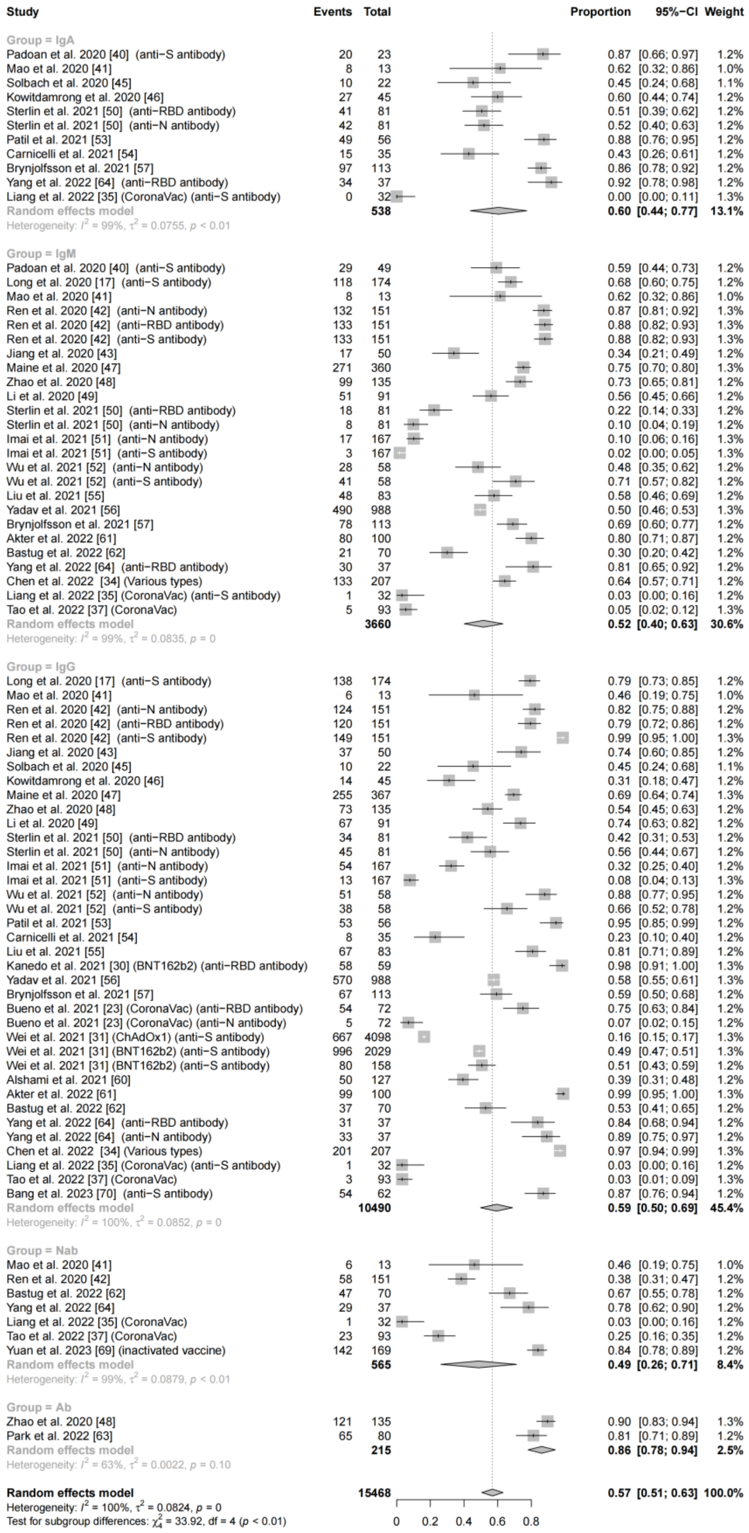

Events: number of participants with detectable antibody levels.

Figure S9. (related to Figure 5) Forest plot of pooled antibody response rates at 15-21 days across antibody types.

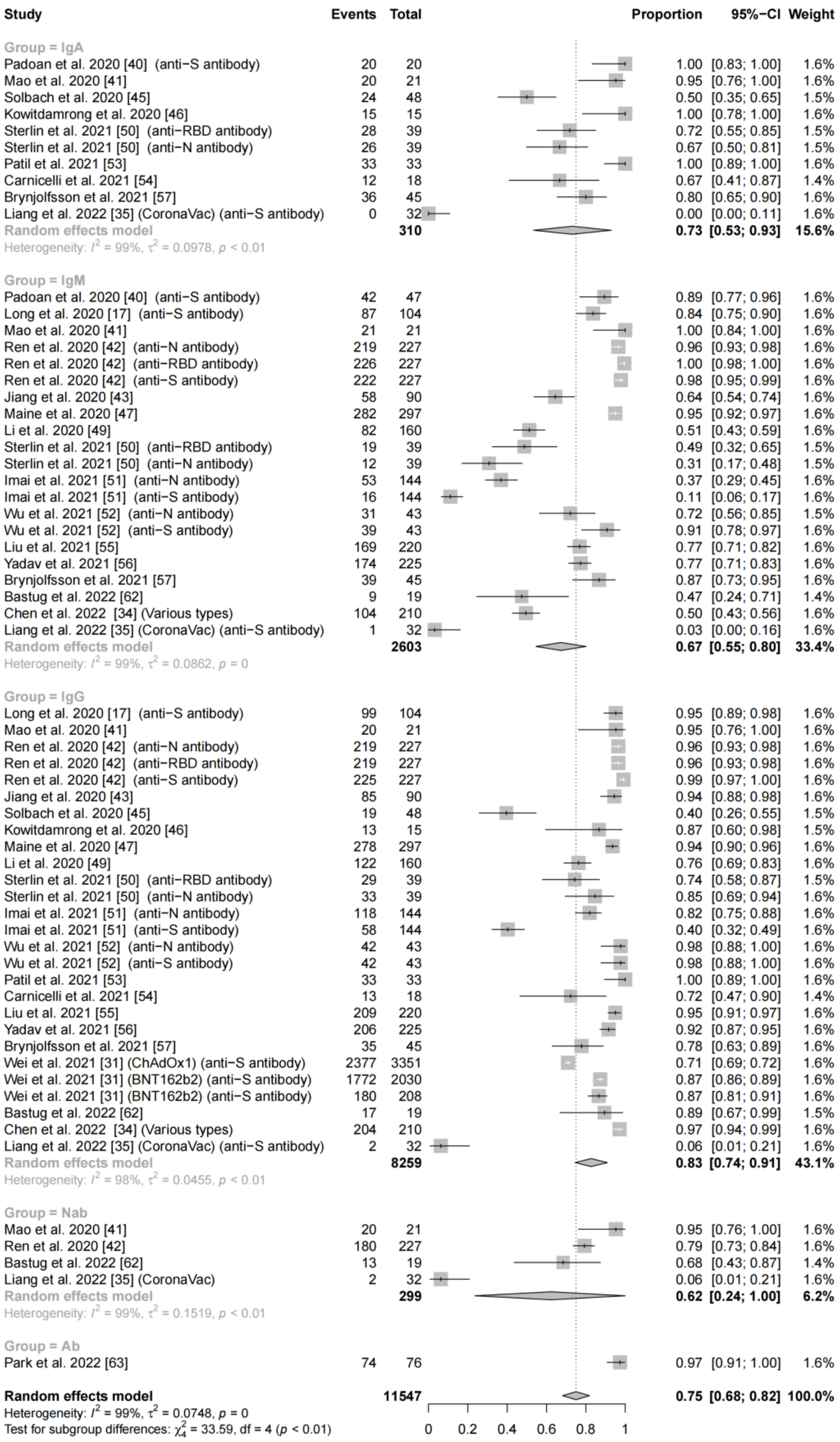

Events: number of participants with detectable antibody levels.

Figure S10. (related to Figure 5) Forest plot of pooled antibody response rates at 22 days -1 month across antibody types.

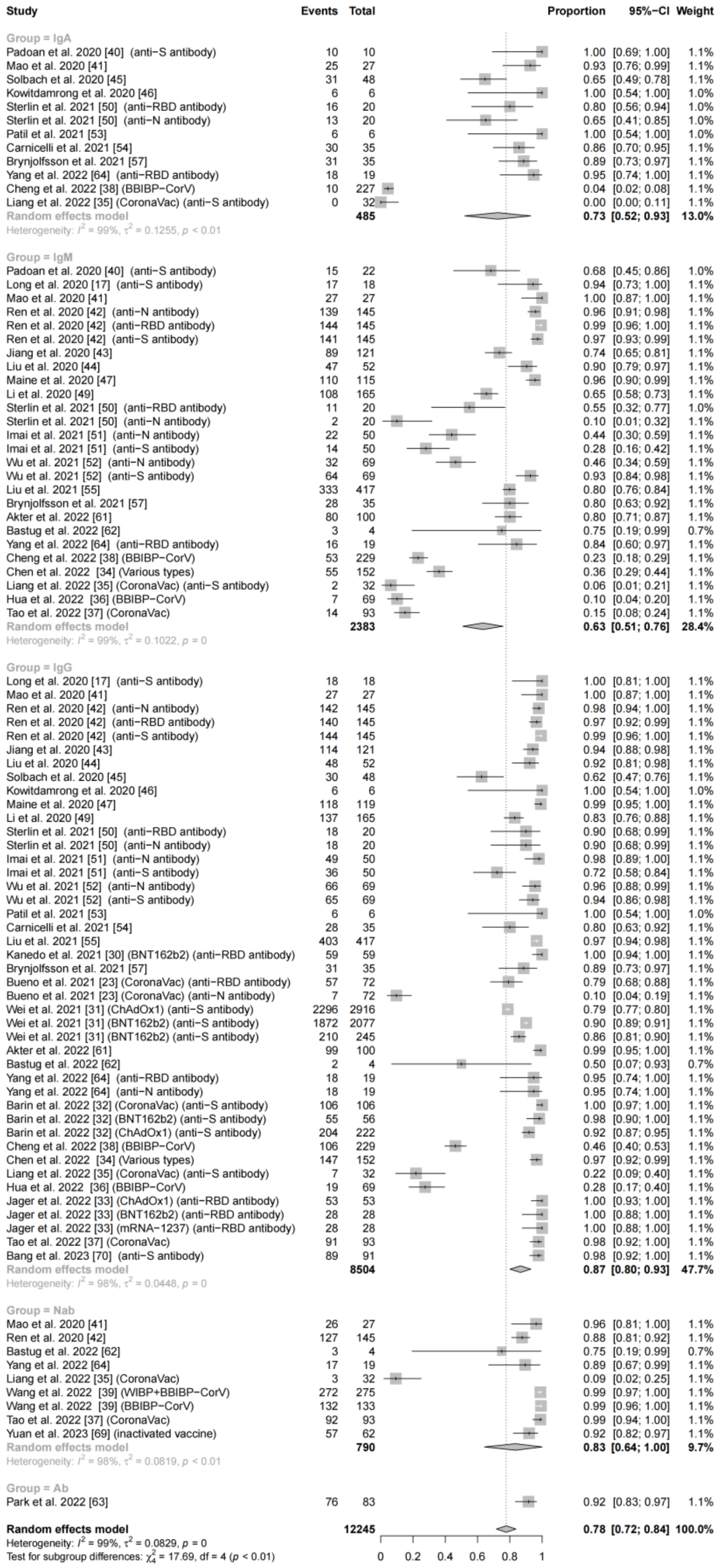

Events: number of participants with detectable antibody levels.

Figure S11. (related to Figure 5) Forest plot of pooled antibody response rates at 1-2 months across antibody types.

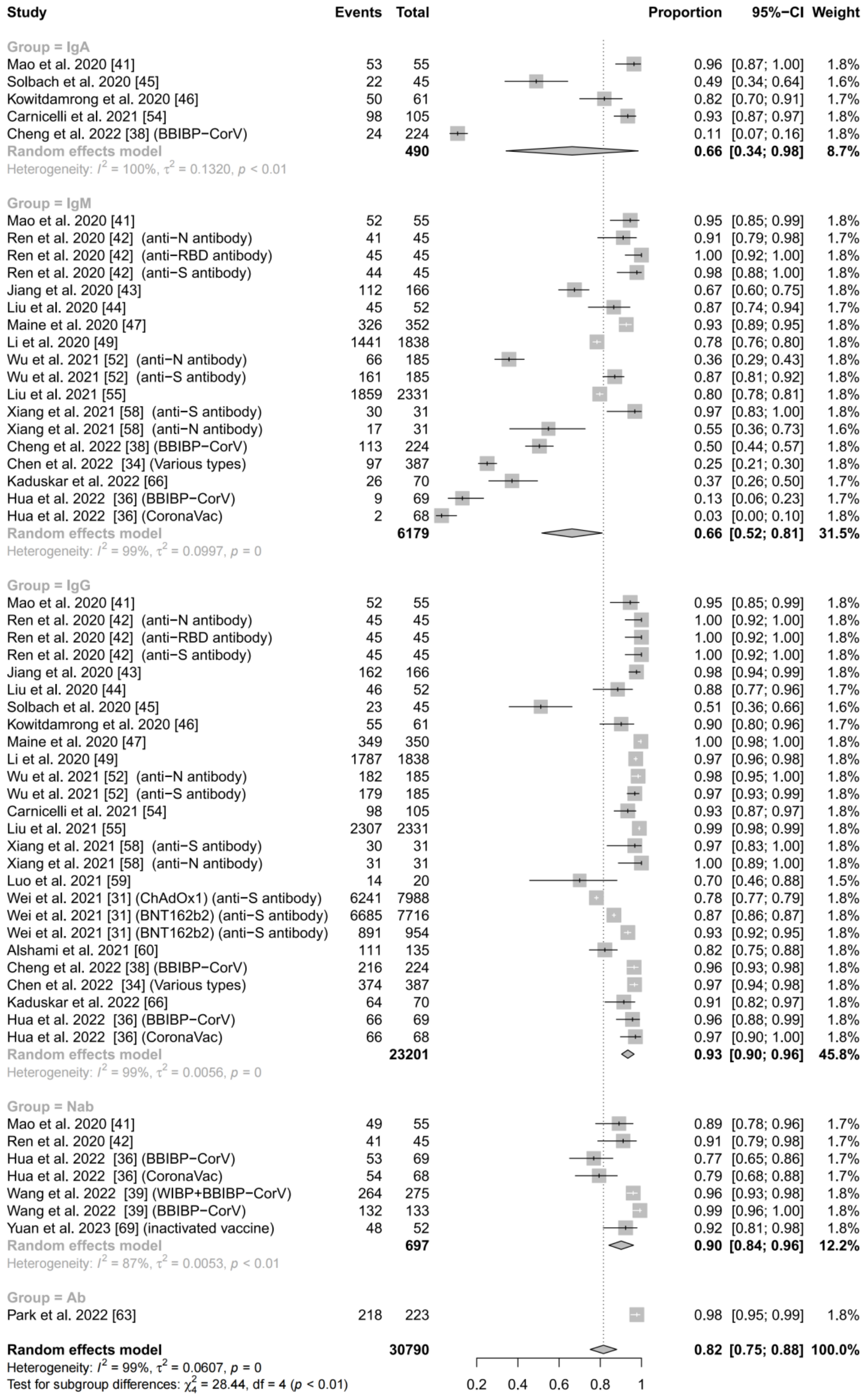

Events: number of participants with detectable antibody levels.

Figure S12. (related to Figure 5) Forest plot of pooled antibody response rates at 2-3 months across antibody types.

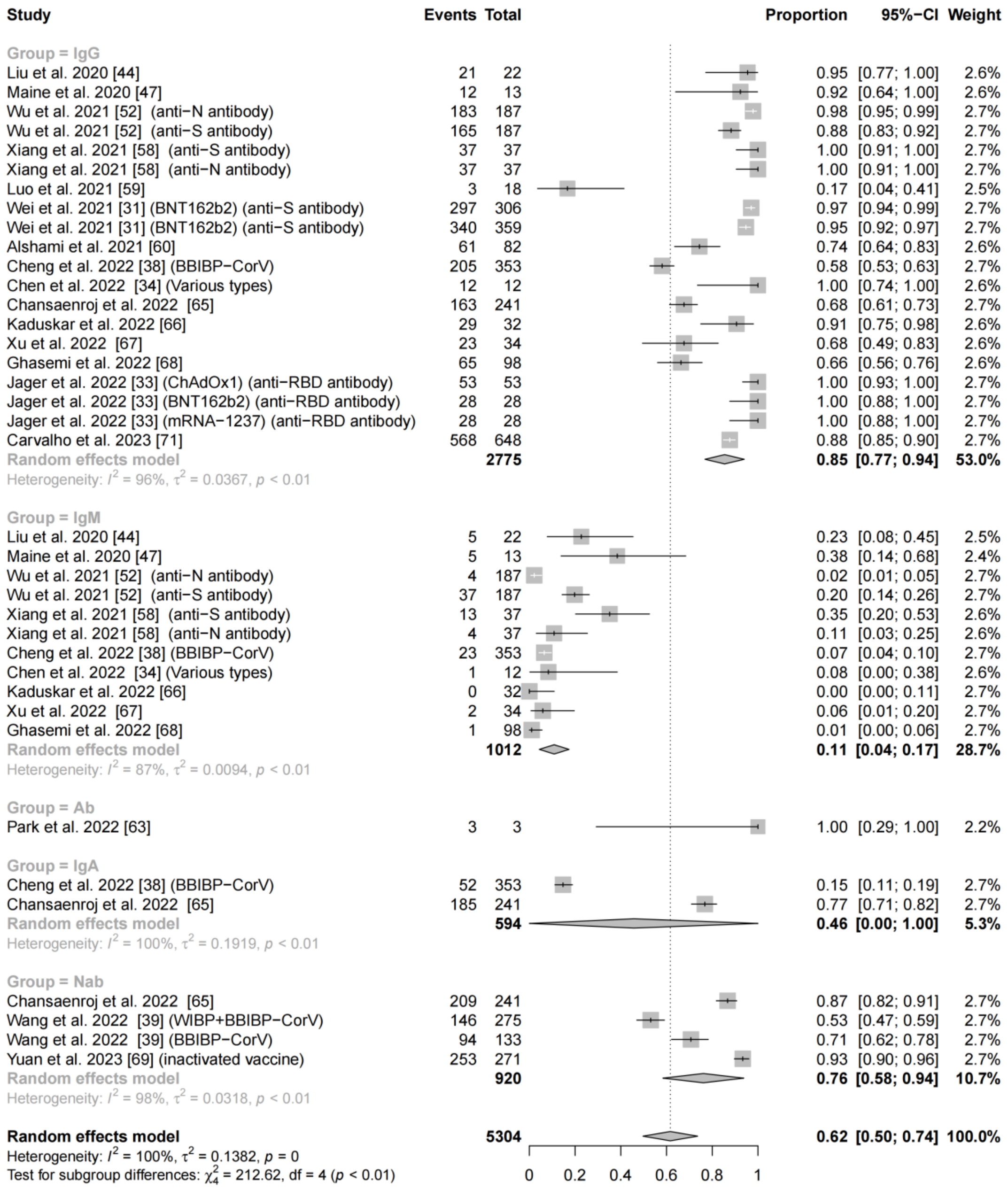

Events: number of participants with detectable antibody levels.

Figure S13. (related to Figure 5) Forest plot of pooled antibody response rates at 3-6 months across antibody types.

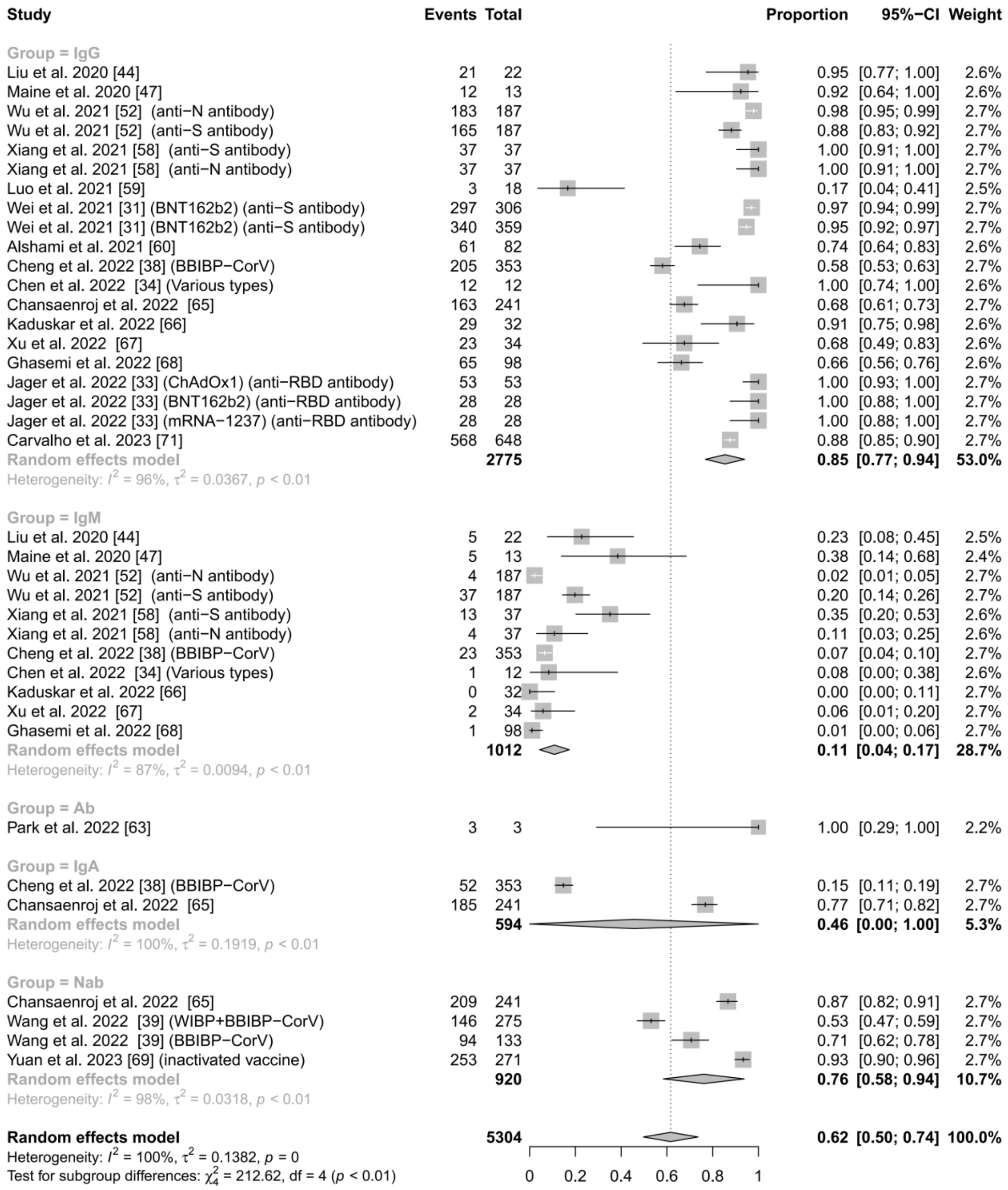

Events: number of participants with detectable antibody levels.

Figure S14. (related to Figure 5) Forest plot of pooled antibody response rates after 6 months across antibody types.

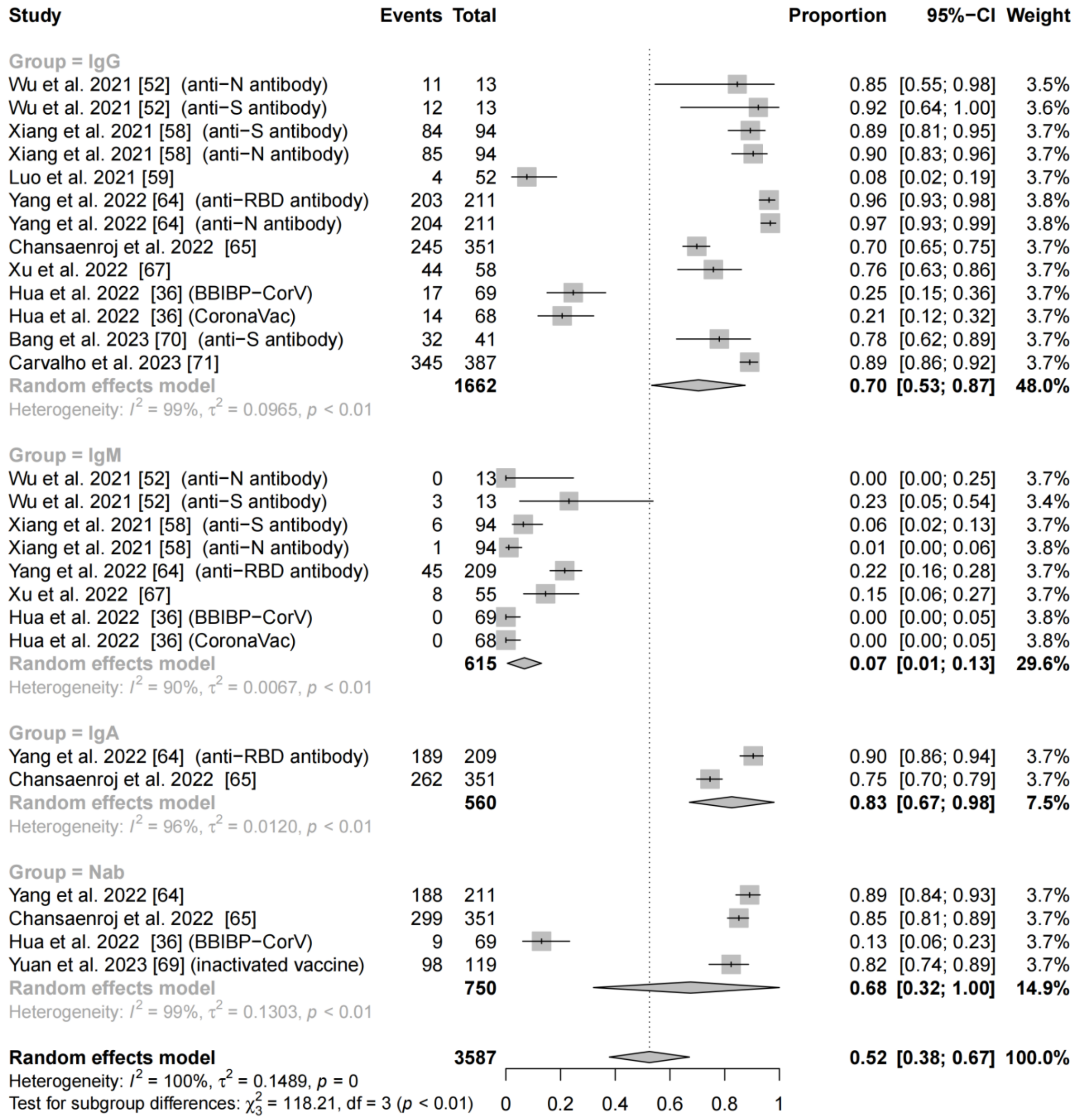

Events: number of participants with detectable antibody levels.

Figure S15. (related to Figure 2) Sensitivity analysis: excluding each study one at a time.

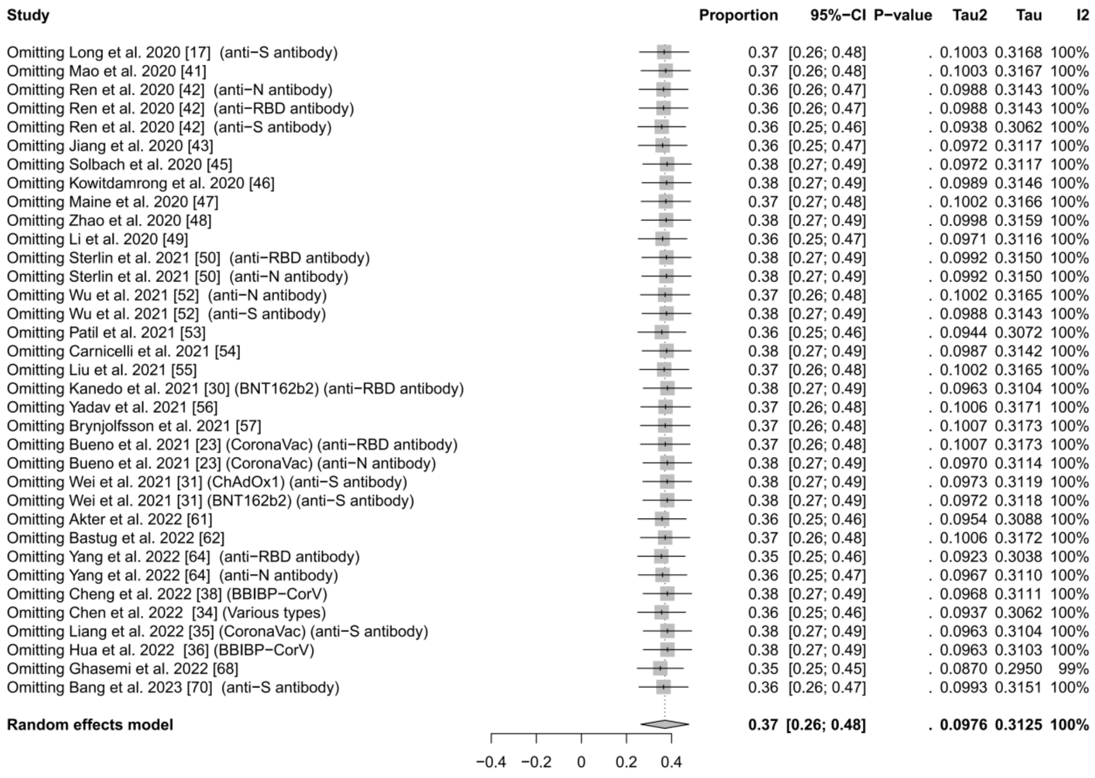

Figure S16. (related to Figure 4) Sensitivity analysis: excluding each study one at a time.

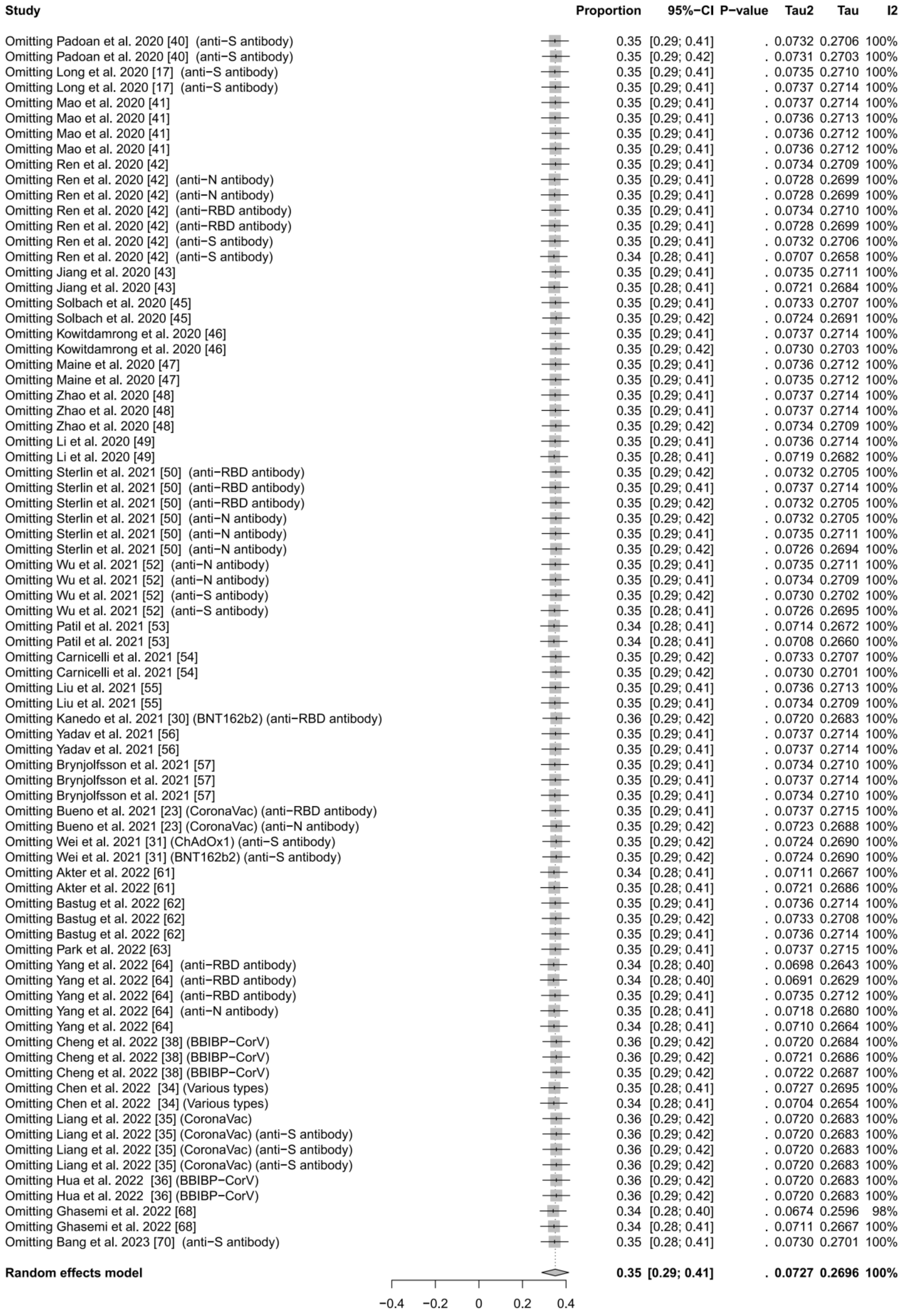

Figure S17. Funnel plots of publication bias and results of Egger's test.

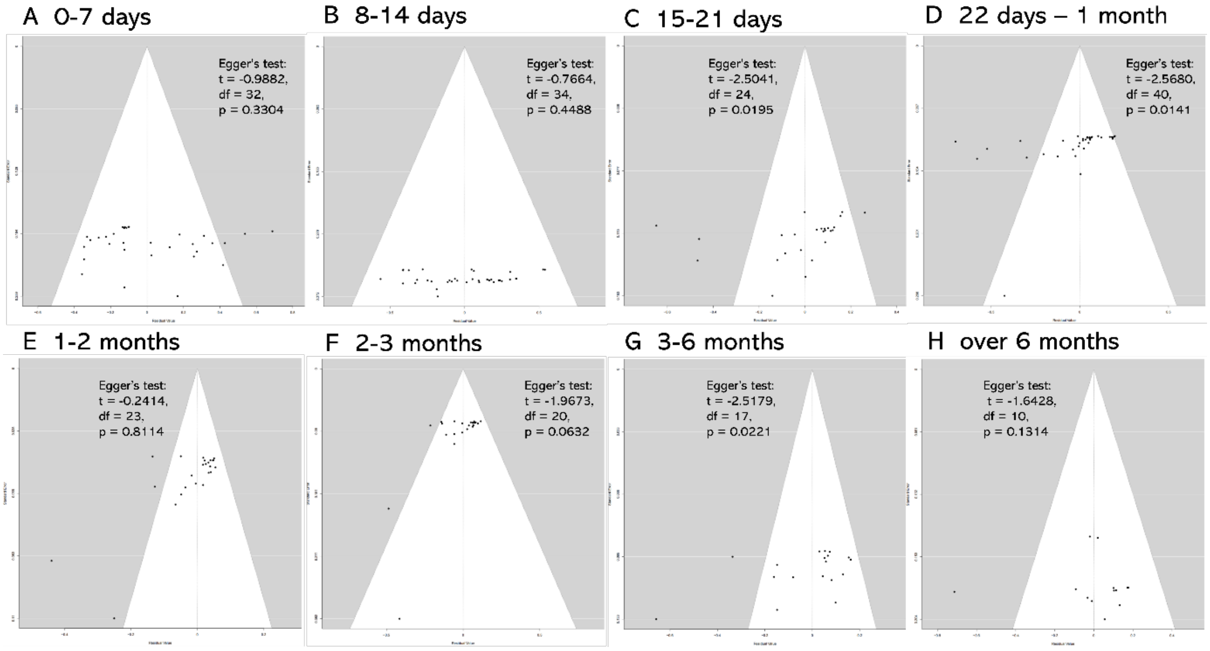

Supplement: Supplementary file 1 — Additional file 1: Appendix. [file 13643_2024_2597_MOESM1_ESM.pdf]
